# Different incidences of diabetic retinopathy requiring treatment since diagnosis according to the course of diabetes diagnosis: a retrospective cohort study

**DOI:** 10.1038/s41598-023-37551-w

**Published:** 2023-06-29

**Authors:** Takehiro Sugiyama, Ayako Yanagisawa-Sugita, Hirokazu Tanaka, Noriko Ihana-Sugiyama, Kenjiro Imai, Mitsuru Ohsugi, Kohjiro Ueki, Nanako Tamiya, Yasuki Kobayashi

**Affiliations:** 1 Diabetes and Metabolism Information Center, Research Institute, National Center for Global Health and Medicine, 1-21-1 Toyama, Shinjuku-ku, Tokyo, 162-8655 Japan; 2 Institute for Global Health Policy Research, Bureau of International Health Cooperation, National Center for Global Health and Medicine, 1-21-1 Toyama, Shinjuku-ku, Tokyo, 162-8655 Japan; 3 Department of Health Services Research, Institute of Medicine, University of Tsukuba, 1-1-1 Tenno-dai, Tsukuba, Ibaraki 305-8575 Japan; 4 Department of Public Health/Health Policy, Graduate School of Medicine, The University of Tokyo, 7-3-1 Hongo, Bunkyo-ku, Tokyo, 113-0033 Japan; 5 Division of Population Data Science, Institute for Cancer Control, National Cancer Center, 5-1-1 Tsukiji, Chuo-ku, Tokyo, 104-0045 Japan; 6 Department of Diabetes, Endocrinology and Metabolism, National Center for Global Health and Medicine, 1-21-1 Toyama, Shinjuku-ku, Tokyo, 162-8655 Japan; 7 Diabetes Research Center, Research Institute, National Center for Global Health and Medicine, 1-21-1 Toyama, Shinjuku-ku, Tokyo, 162-8655 Japan

**Keywords:** Diabetes complications, Population screening

## Abstract

We aimed to estimate the cumulative incidence of treatment-requiring diabetic retinopathy since clinical diagnosis of diabetes based on the course of diagnosis in a retrospective cohort study using Japan’s medical claims and health checkup data (JMDC Claims Database; 2009–2020). We included patients whose diabetes was first diagnosed at medical facilities (hospitals/clinics). We grouped them by health checkup participation before diagnosis, health checkup results, and antidiabetic medication promptly after the diagnosis. The incidence of treatment-requiring diabetic retinopathy (laser photocoagulation, intraocular injection, or vitrectomy) was compared among the groups. Of 126,696 patients, those who started an antidiabetic medication promptly after diabetes diagnosis without a recent health checkup faced the highest risk of treatment-requiring diabetic retinopathy (1-/5-year cumulative incidence: 3.1%/6.0%). This increased risk was consistently observed across various analyses, including the Cox proportional hazard model, sensitivity analysis restricting to those with an eye examination, and sensitivity analysis using vitrectomy as the outcome. Among patients with HbA1c ≥ 6.5% at recent health checkups, those who promptly started an antidiabetic medication had a higher risk (1.4%/3.8%) than those who did not (0.7%/2.7%). Taking the information about the course of diabetes diagnosis is important to manage risk stratification for diabetic retinopathy appropriately.

## Introduction

Diabetes is an important public health threat that affects 537 million people worldwide^1^. It increases the risk of microangiopathies, such as acute coronary syndrome, stroke, peripheral arterial diseases, and risk for microangiopathy, including retinopathy. A previous study indicated that age, age at diagnosis, and duration of diabetes were independently associated with future macroangiopathy, whereas the only duration of diabetes was independently associated with the incidence of future microangiopathy^2^.

Retinopathy often develops approximately 5 years after diabetes diagnosis^3^; however, some patients are diagnosed with retinopathy before or after diagnosis of diabetes, which implies that retinopathy may develop before clinical diagnosis of diabetes^4,5^. The presumed duration between the actual but unrecognized incidence of diabetes and the clinical diagnosis of diabetes can vary depending on how diabetes is diagnosed, thus indicating a potential association between the course of diabetes diagnosis and the incidence of retinopathy following the clinical diagnosis of diabetes, even after accounting for the duration of diabetes since diagnosis. Nonetheless, limited research has investigated the association between how diabetes was diagnosed and the incidence of microangiopathy.

The present study estimated the cumulative incidence of treatment-requiring diabetic retinopathy since the clinical diagnosis of diabetes, considering how diabetes was diagnosed. Furthermore, it aimed to investigate differences in the risk of diabetic retinopathy by employing medical claims and health checkup data in Japan.

## Methods

### Data sources and study population

The present retrospective cohort study was conducted using a combination of medical claims and health checkup data in Japan. Data were collected from part of the JMDC Claims Database^6^, which includes medical claims and health checkups information from employer-sponsored health insurance. Employer-sponsored health insurance mainly covers employees of large companies and their dependents. Medical claims data had disease name, medication, and medical activity, including laboratory tests performed but not the results. Health checkup information was derived from regular health checkups for employees based on the Industrial Safety and Health Act and specific health checkups for those aged 40–74 years based on the Act on Assurance of Medical Care for Elderly People. Generally, the participation rate of health checkups was higher among employees than among dependents because employers were responsible for higher participation rates of their employees.

**Figure 1:**
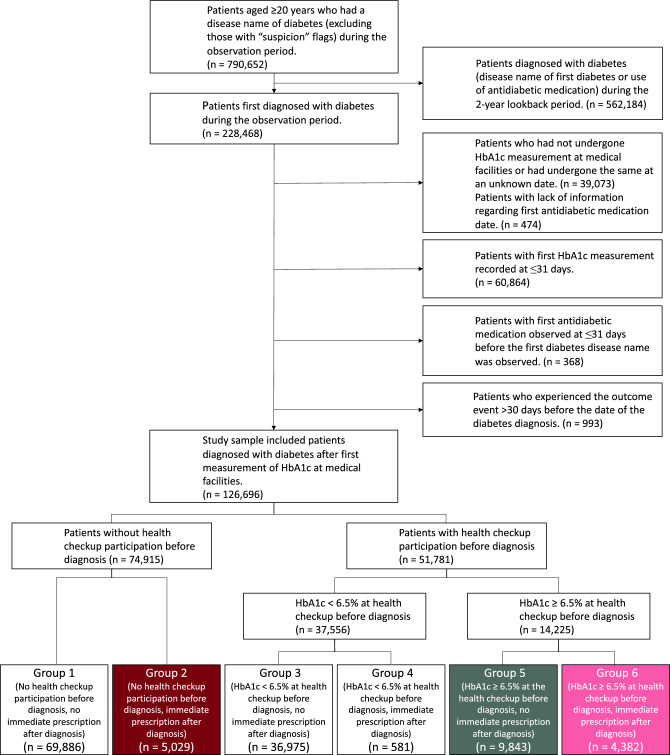
Flow chart of study sample selection and categorization. We were especially interested in the three groups shown in color: Group 2 (no health checkup participation before diagnosis, prompt antidiabetic medication after diagnosis), Group 5 (HbA1c ≥ 6.5% at health checkup before diagnosis, no prompt antidiabetic medication after diagnosis), and Group 6 (HbA1c ≥ 6.5% at health checkup before diagnosis, prompt antidiabetic medication after diagnosis). The colors in this figure are consistent with those in Figs. 2 and 3.

The present study examined patients with diabetes first diagnosed at medical facilities (hospitals/clinics) during the observation period. An outline of the patient selection and data collection is shown in Supplementary Fig. S1; a flow chart of patient selection is shown in Fig. 1. We included patients aged ≥ 20 years who had a disease name diabetes (excluding those with “suspicion” flags) during the observation period. Excluding those diagnosed with diabetes (disease name of first diabetes or antidiabetic medication) during the 2-year lookback period ensured that we only selected patients first diagnosed with diabetes during the observation period. We excluded patients lacking HbA1c measurements at medical facilities, those with unknown dates of HbA1c measurement, and those lacking information about their first antidiabetic medication date. We also excluded patients whose first HbA1c measurement was observed for > 30 days after diagnosis of diabetes because diabetes diagnosis was made using HbA1c measurement, and it was difficult to distinguish the date of diagnosis if multiple HbA1c measurements without diabetes disease names existed. We also excluded those whose first antidiabetic medication was observed for > 30 days before the disease name of first diabetes. Finally, data were collected, excluding patients whose outcome event had already occurred for > 30 days before the diabetes diagnosis.

### Measurements

#### Clinical diagnosis of diabetes

Clinical diagnosis of diabetes was made based on the disease name of diabetes or the use of antidiabetic medication. The disease name of diabetes was defined as E10–14 of the 10th revision of the International Statistical Classification of Diseases and Related Health Problems as stated in the medical claims. Disease name included information about the “start date of treatment” determined by a physician in the medical claims data; we defined the date of the first diagnosis of diabetes based on this information. Antidiabetic medication was defined using the A10 code of the Anatomical Therapeutic Chemical classification system, excluding A10X (aldose reductase inhibitor) and 0.2 mg Voglibose tablet (which may have been used to treat impaired glucose tolerance).

#### Outcome variables

Diabetic retinopathy requiring treatment was determined from the intervention for diabetic retinopathy and consisted of laser photocoagulation, intraocular injection, and vitrectomy. The outcome event was the first appearance of procedure codes K276 for laser photocoagulation, G016 for intraocular injection, and K280, K280-2, or K281 for vitrectomy in the medical claims database. When information about the “day” of treatment was not available (< 8% of all treatments), the 15th of the month was used.

#### Main predictor

We classified the patients into six groups according to health checkup participation, health checkup results, and antidiabetic medication promptly after the clinical diagnosis of diabetes. We classified the patients into two groups according to whether they had health checkups and HbA1c measurements at a health checkup 6 months before diagnosis (Fig. 1). Participants of a health checkup group were then classified according to HbA1c level at the health checkup (< 6.5%, ≥ 6.5%). The three groups (no participation, HbA1c < 6.5%, and HbA1c ≥ 6.5%) were each dichotomized according to whether they received antidiabetic medication promptly after the diagnosis (within 7 days).

Among the six groups, we were particularly interested in Group 2 (no health checkup participation before diagnosis, prompt antidiabetic medication after diagnosis), Group 5 (HbA1c ≥ 6.5% at health checkup before diagnosis, no prompt antidiabetic medication after diagnosis), and Group 6 (HbA1c ≥ 6.5% at health checkup before diagnosis, prompt antidiabetic medication after diagnosis). Group 1 (no health checkup participation before diagnosis, no prompt antidiabetic medication after diagnosis) and Group 3 (HbA1c < 6.5% at health checkup before diagnosis, no prompt antidiabetic medication after diagnosis) may have included individuals without diabetes due to possible inaccuracy of the disease name in the medical claims data. Group 4 (HbA1c < 6.5% at health checkup before diagnosis, prompt antidiabetic medication after diagnosis) included small numbers, and it was difficult to interpret the situation.

#### Other variables

HbA1c measurements at medical facilities were detected from medical claims.

The month of birth, gender, employees/dependent variable, and the observation period for each beneficiary (the term of insurance while data were available) were taken from the ledger of beneficiaries; age was calculated from the month of birth and the date of diabetes diagnosis; accurately, the age was on the last day in the previous month of the diabetes diagnosis.

Type 1 diabetes at diagnosis was defined as E10 of ICD-10 within 1 month from the clinical diagnosis of diabetes.

We also prepared hypertension drug use and dyslipidemia drug use. Because hypertension drugs can be used for other diseases (e.g., heart failure, arrhythmia), we defined hypertension drugs use as having both a drug used for hypertension and a hypertension disease name in the same month. In contrast, we defined dyslipidemia drug use simply as having dyslipidemia drugs. We also made a Charlson Comorbidity Index updated by Quan et al.^7^. We included congestive heart failure, dementia, chronic pulmonary disease, rheumatologic disease, mild liver disease, hemiplegia or paraplegia, renal disease, any malignancy including leukemia and lymphoma, moderate or severe liver disease, and metastatic solid tumor. We did not include diabetes with/without complications because they all had diabetes. The disease name information for AIDS/HIV was intentionally excluded from the database for privacy reasons. For hypertension drugs, dyslipidemia drugs, and the Charlson Comorbidity Index, we observed from 6 months before and 1 month after the time zero.

We prepared a variable about the eye exam within 6 months of diabetes diagnosis for a sensitivity analysis. We defined eye exam with D255, D255-2, D256, D256-2, D256-3, D257. When the “day” information of treatment was not available (< 12% of all treatments), we imputed the date with the 15th of each month.

The definition of each variable is listed in Supplementary Table S1.

### Statistical analyses

After the patient selection, the characteristics of the participants (age at diagnosis, sex, employee/dependent variable, and type 1 diabetes at diagnosis) were collected for each group. We also excluded patients who experienced the outcome event > 30 days before the date of the diabetes diagnosis.

Next, we conducted a survival analysis, setting the date of diabetes diagnosis as time zero and treatment-requiring diabetic retinopathy as the outcome event. Unlike typical survival analyses, we included patients who experienced events just before (within 30 days) or on the same day as the diabetes diagnosis and set their event time as 0.5 days. The Kaplan–Meier method calculated the group-stratified cumulative incidence of treatment-requiring diabetic retinopathy since diabetes diagnosis. Since we were particularly interested in Groups 2, 5, and 6, we created a figure focusing on these three groups and describing all six groups. The groups were observed until the date of the event, the end of the observation period, or 5 years from time zero, whichever occurred first. Differences between the groups were examined using the log-rank test. We also adjusted for covariates (10-year age categories, sex, employee/dependent variable, and type 1 diabetes) using the Cox proportional hazards model. We also calculated the incidence rates of outcome events according to the characteristics.

Because retinopathy treatment should occur after detection during the eye exam, we focused on those who had an eye examination between 6 months before and 1 year after the diabetes diagnosis as a sensitivity analysis. An eye examination after diabetes diagnosis was recommended in the clinical guideline. Another sensitivity analysis used only vitrectomy as the outcome event as it is more invasive than the others, and laser photocoagulation prevents retinopathy progression and avoids vitrectomy or blindness. We repeated the calculation of the cumulative incidence of a treatment since diabetes diagnosis focusing on the three groups (Groups 2, 5, and 6) using the Kaplan–Meier method in the sensitivity analyses.

We used Stata 15.1 (StataCorp, College Station, Texas, USA) for statistical analysis and data management. *P*-values < 0.05 were considered statistically significant using two-sided hypothesis tests. Because the degree of missingness was low, we conducted complete-case analyses.

Patients and the public were not involved in any way.

### Ethics approval

All methods were carried out in accordance with relevant guidelines and regulations. Data were anonymized; it was impossible to re-identify patients in the study. Therefore, opt-out or opt-in was impossible and not required from the institutional review board in accordance with the ethical guidelines. The National Center for Global Health and Medicine approved the present observational study, including the waiver of the need for informed consent (NCGM-G-002096-06).

### Prior presentations

We presented a part of the present study at the 32nd Annual Scientific Meeting of the Japan Epidemiological Association (January 2022, online meeting).

## Results

A flowchart of the present study is shown in Fig. 1. We examined 126,696 patients diagnosed with diabetes after their first HbA1c measurement at medical facilities. We further divided these patients into six groups. Supplementary Table S2 shows the numbers and proportions of those excluded due to outcome events experienced > 30 days before diabetes diagnosis. The proportions of patients excluded in Groups 2, 5, and 6 were 0.4%, 0.6%, and 0.6%, respectively.

The patient’s characteristics are shown in Table 1. Groups 5 and 6 had more males than Group 2 (77.7% and 80.8% vs. 65.6%, respectively). Similarly, Groups 5 and 6 had more employees than Group 2 (77.7% and 80.8%, respectively, vs. 71.7%). Group 2 contained more patients with type 1 diabetes than Groups 5 and 6 (3.3% vs. 0.7% and 1.1%, respectively).

**Table 1:** Characteristics of the patients according to the study groups.

	Group 1	Group 2	Group 3	Group 4	Group 5	Group 6	*P*-value *
n	69,886	5029	36,975	581	9843	4382	
Age at baseline [mean (SD) in years]	47.6 (12.1)	48.9 (11.3)	50.1 (9.4)	51.2 (9.1)	51.3 (8.6)	50.3 (8.3)	< 0.001
10-year age category at baseline							
< 30, n (%)	6047 (8.7%)	262 (5.2%)	899 (2.4%)	13 (2.2%)	108 (1.1%)	49 (1.1%)	< 0.001
30–39, n (%)	12,681 (18.1%)	827 (16.4%)	3549 (9.6%)	37 (6.4%)	673 (6.8%)	334 (7.6%)	
40–49, n (%)	18,154 (26.0%)	1438 (28.6%)	12,477 (33.7%)	184 (31.7%)	3251 (33.0%)	1611 (36.8%)	
50–59, n (%)	20,951 (30.0%)	1577 (31.4%)	14,101 (38.1%)	250 (43.0%)	4166 (42.3%)	1787 (40.8%)	
60–69, n (%)	10,412 (14.9%)	788 (15.7%)	5464 (14.8%)	82 (14.1%)	1511 (15.4%)	572 (13.1%)	
≥ 70, n (%)	1641 (2.3%)	137 (2.7%)	485 (1.3%)	15 (2.6%)	134 (1.4%)	29 (0.7%)	
Sex							
Male, n (%)	39,143 (56.0%)	3301 (65.6%)	24,690 (66.8%)	450 (77.5%)	7649 (77.7%)	3542 (80.8%)	< 0.001
Female, n (%)	30,743 (44.0%)	1728 (34.4%)	12,285 (33.2%)	131 (22.5%)	2194 (22.3%)	840 (19.2%)	
Employee/dependent							
Employee, n (%)	46,543 (66.6%)	3604 (71.7%)	30,008 (81.2%)	510 (87.8%)	8630 (87.7%)	3902 (89.0%)	< 0.001
Dependent, n (%)	23,343 (33.4%)	1425 (28.3%)	6967 (18.8%)	71 (12.2%)	1213 (12.3%)	480 (10.0%)	
Type of diabetes							
Type 1, n (%)	223 (0.3%)	167 (3.3%)	74 (0.2%)	19 (3.3%)	69 (0.7%)	49 (1.1%)	< 0.001
Type 2 and others, n (%)	69,663 (99.7%)	4862 (96.7%)	36,901 (99.8%)	562 (96.7%)	9774 (99.3%)	4333 (98.9%)	
Hypertension drugs, n (%)							
No	52,780 (75.5%)	3481 (69.2%)	27,842 (75.3%)	349 (60.1%)	7458 (75.8%)	3158 (72.1%)	< 0.001
Yes	17,106 (24.5%)	1548 (30.8%)	9133 (24.7%)	232 (39.9%)	2385 (24.2%)	1224 (27.9%)	
Dyslipidemia drugs, n (%)							
No	57,164 (81.8%)	3782 (75.2%)	28,956 (78.3%)	344 (59.2%)	7990 (81.2%)	3232 (73.8%)	< 0.001
Yes	12,722 (18.2%)	1247 (24.8%)	8019 (21.7%)	237 (40.8%)	1853 (18.8%)	1150 (26.2%)	
CCI category, n (%)							
< 3	59,119 (84.6%)	4458 (88.6%)	31,097 (84.1%)	508 (87.4%)	9063 (92.1%)	4052 (92.5%)	< 0.001
≥ 3	10,767 (15.4%)	571 (11.4%)	5878 (15.9%)	73 (12.6%)	780 (7.9%)	330 (7.5%)	

CCI, Charlson comorbidity index.

*P-values are calculated using chi-squared tests for categorical variables and ANOVA for continuous age.

**Figure 2:**
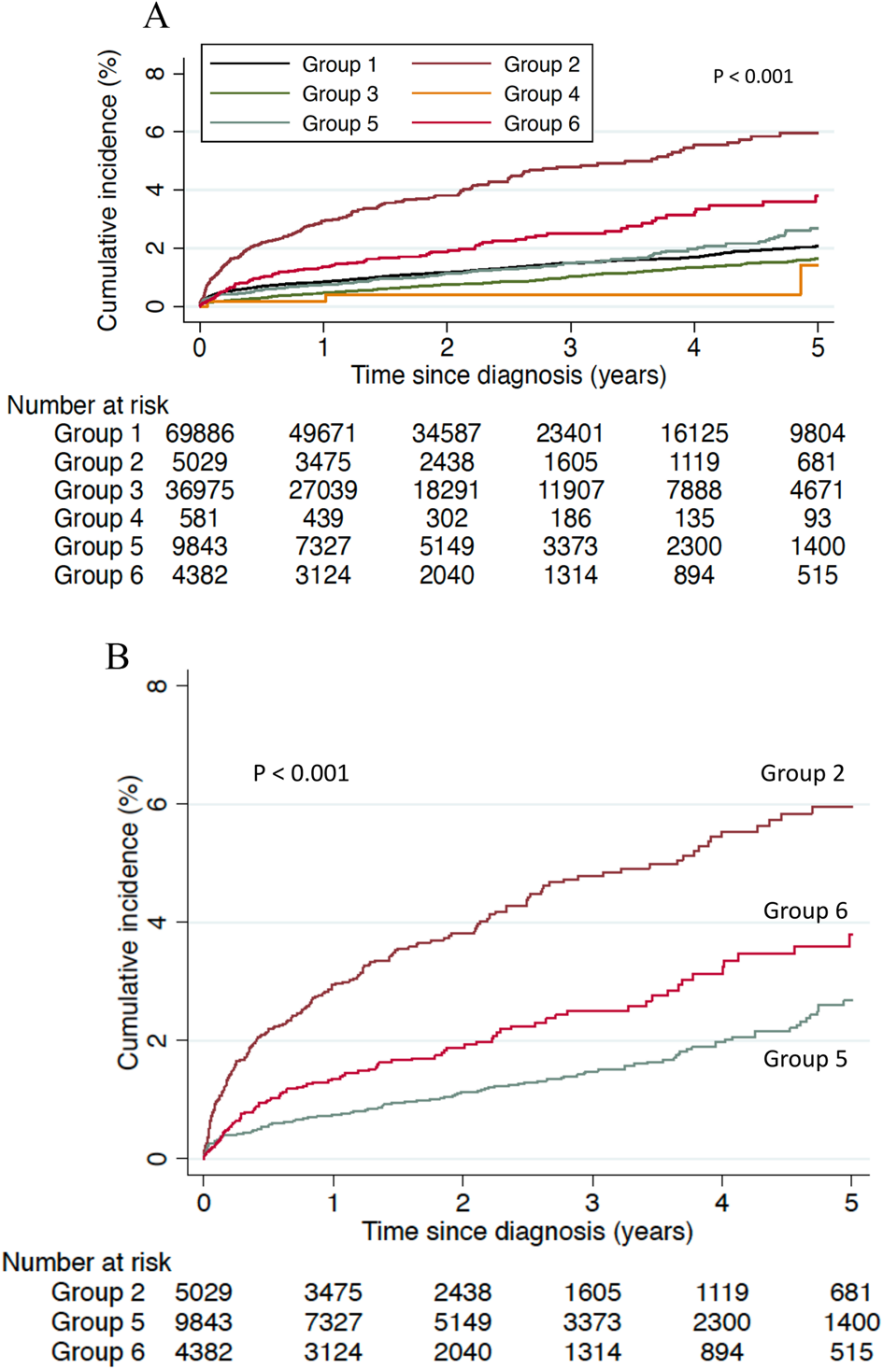
Cumulative incidence of treatment-requiring diabetic retinopathy since diabetes diagnosis according to how diabetes was diagnosed. (**A**) Kaplan–Meier curves of all six groups. P < 0.001 by a log-rank test. (**B**) Kaplan–Meier curves of the interested three groups only (Groups 2, 5, 6). P < 0.001 by a log-rank test. Group 1: No health checkup participation before diagnosis, no prompt antidiabetic medication after diagnosis. Group 2: No health checkup participation before diagnosis, prompt antidiabetic medication after diagnosis. Group 3: HbA1c < 6.5% at the health checkup before diagnosis, no prompt prescription after diagnosis. Group 4: HbA1c < 6.5% at health checkup before diagnosis, prompt prescription after diagnosis. Group 5: HbA1c ≥ 6.5% at the health checkup before diagnosis, no prompt prescription after diagnosis. Group 6: HbA1c ≥ 6.5% at health checkup before diagnosis, prompt prescription after diagnosis.

The incidence rates of outcome events according to the characteristics are shown in Supplementary Table S3. The Kaplan–Meier curves of all six groups are shown in Fig. 2A. The Kaplan–Meier curves of the three groups of interest (Groups 2, 5, and 6) are shown in Fig. 2B. The curves were convex upward, especially in Group 2. The cumulative incidences of diabetic retinopathy requiring treatment in Groups 2, 5, and 6 were 3.1%, 0.7%, and 1.4% after 1 year, and 6.0%, 2.7%, and 3.8%, respectively, after 5 years. The curves were significantly different (Group 2 vs. Group 5: *P* < 0.001; Group 2 vs. Group 5: *P* < 0.001; Group 5 vs. Group 6: *P* < 0.001). The Kaplan–Meier curves of outcome events according to other characteristics are shown in Supplementary Fig. S2. The Kaplan–Meier curves of outcome events according to characteristics among Groups 2, 5, and 6 are shown in Supplementary Fig. S3. The relationship between sex or employee/dependent and the outcome events appeared to be modified by how diabetes was diagnosed.

The Cox proportional hazards model results are shown in Table 2. Treatment-requiring diabetic retinopathy was more frequent in Group 2 than in any other group. Older age, male sex, dependent rather than employee, type 1 diabetes diagnosis, use of hypertension drugs, and no use of dyslipidemia drugs were also associated with a higher incidence in the Cox proportional hazard model.

**Table 2:** Risk factors for treatment-requiring diabetic retinopathy: results from Cox proportional hazard model (N = 126,696).

	Hazard ratio (95% CI)	*P*-value
Group		
1	0.33 (0.28–0.39)	< 0.001
2	1 (reference)	
3	0.22 (0.18–0.26)	< 0.001
4	0.12 (0.04–0.38)	< 0.001
5	0.33 (0.27–0.42)	< 0.001
6	0.55 (0.43–0.71)	< 0.001
10-year age category at baseline		
< 30	1 (reference)	
30–39	1.45 (0.91–2.32)	0.12
40–49	3.02 (1.96–4.65)	< 0.001
50–59	5.22 (3.40–8.01)	< 0.001
60–69	6.10 (3.95–9.43)	< 0.001
≥ 70	6.49 (3.90–10.79)	< 0.001
Sex		
Male	1 (reference)	
Female	0.84 (0.70–1.01)	0.06
Employee/dependent		
Employee	1 (reference)	
Dependent	1.76 (1.46–2.12)	< 0.001
Type of diabetes		
Type 1	1.99 (1.23–3.23)	0.005
Type 2 and others	1 (reference)	
Hypertension drugs		
No	1 (reference)	
Yes	1.39 (1.25–1.55)	< 0.001
Dyslipidemia drugs		
No	1 (reference)	
Yes	0.86 (0.76–0.97)	0.01
CCI category		
< 3	1 (reference)	
≥ 3	1.03 (0.90–1.18)	0.66

CCI, Charlson comorbidity index.

**Figure 3:**
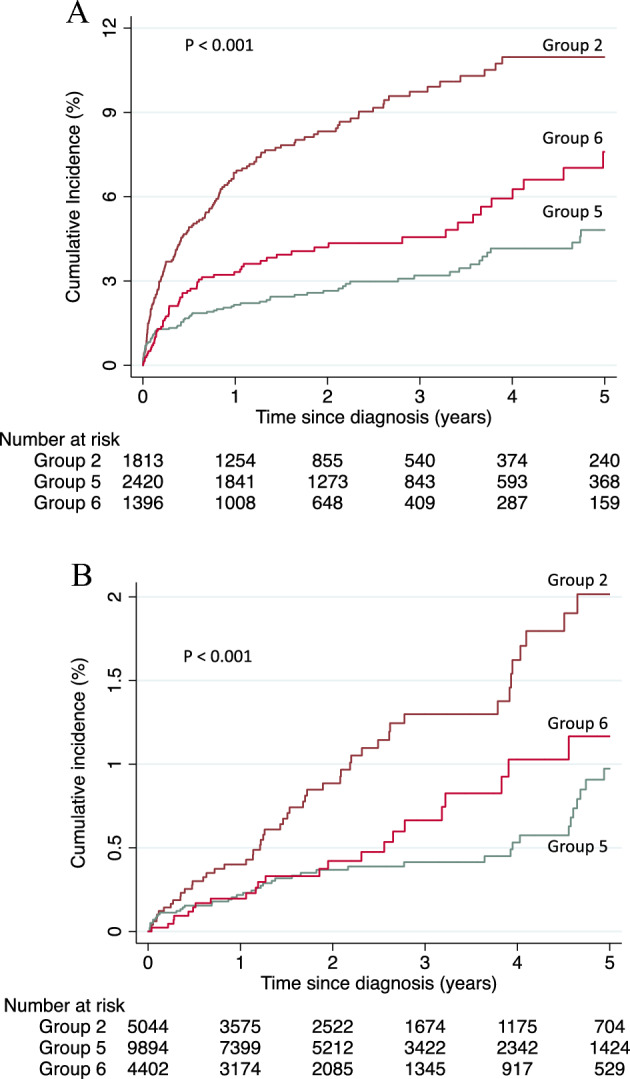
Sensitivity analyses of the present study. (**A**) Patients who underwent an eye examination between 6 months before and 1 year after the diabetes diagnosis. P < 0.001 by a log-rank test. (**B**) Analysis using only vitrectomy as the outcome event. P < 0.001 by a log-rank test. Group 2: No health checkup participation before diagnosis, prompt antidiabetic medication after diagnosis. Group 5: HbA1c ≥ 6.5% at the health checkup before diagnosis, no prompt prescription after diagnosis. Group 6: HbA1c ≥ 6.5% at health checkup before diagnosis, prompt prescription after diagnosis.

Figure 3A shows the sensitivity analysis results for those who underwent an eye examination between 6 months before and 1 year after the diabetes diagnosis. The incidence of retinopathy within 1 year after the diagnosis was elevated, and the differences among the groups were maintained. The cumulative incidences of diabetic retinopathy requiring treatment in Groups 2, 5, and 6 were 6.9%, 2.2%, and 3.3% after 1 year and 11.0%, 4.8%, and 7.6% after 5 years, respectively (Group 2 vs. Group 5: *P* < 0.001; Group 2 vs. Group 6: *P* < 0.001; Group 5 vs. Group 6: *P* = 0.009).

Figure 3B shows the sensitivity analysis results using only vitrectomy as the outcome event. The incidence rate of outcome events was relatively low; the cumulative incidences of diabetic retinopathy requiring treatment in Groups 2, 5, and 6 were 0.4%, 0.2%, and 0.2% after 1 year, and 2.0%, 1.0%, and 1.2% after 5 years, respectively. The incidence rate of Group 2 was significantly higher than those of Group 5 (*P* < 0.001) and Group 6 (*P* = 0.01).

## Discussion

The present study showed that patients with the same duration of diabetes have a heterogeneous risk of diabetic retinopathy requiring treatment. Notably, patients in Group 2, who did not participate in health checkups prior to diagnosis but promptly received antidiabetic medication after diagnosis, exhibited the highest risk. It is uncommon to prescribe an antidiabetic medication for patients first diagnosed with diabetes unless their glycemic levels are relatively high. Therefore, Group 2 may include patients who were prescribed antidiabetic medications based on the first measurement of HbA1c levels. Among patients with HbA1c levels ≥ 6.5% at health checkups before diagnosis, those with prompt antidiabetic medication after diagnosis (Group 6) had a higher risk than those without prompt antidiabetic medication (Group 5). The sensitivity analyses showed that the risk heterogeneity was robust compared with those who underwent an eye examination between 6 months before and 1 year after the diabetes diagnosis or change of outcome event of only vitrectomy. To the best of our knowledge, this is the first study to show that the future risk of diabetic retinopathy after diabetes diagnosis may vary according to how diabetes was diagnosed.

Several prior studies have investigated risk factors for diabetic retinopathy. Our previous study showed that older age, male sex, being dependency status (rather than being an employee), use of insulin, higher Charlson comorbidity index, and lack of dyslipidemia drugs use were associated with a higher risk of diabetic retinopathy requiring treatment among those who used antidiabetic medication^8^. Another recent study investigated the incidence of proliferative diabetic retinopathy at 5 years after the diagnosis of type 2 diabetes^9^. Other studies have examined the onset of retinopathy by plotting the cumulative incidence of retinopathy after the diabetes diagnosis. For example, Spijkerman et al.^10^ investigated differences in retinopathy at the time of type 2 diabetes diagnosis based on targeted screening versus general practice detection. They found that the prevalence of diabetic retinopathy was 7.6% in patients whose diabetes was detected during targeted screening compared to 1.9% of patients whose diabetes was detected by general practitioners. Although this association appears contradictory to the findings of our present study, it should be noted that screening interventions were employed in both settings, which differed from the approach in our study. Patients whose diabetes was detected during targeted screening may have included those whose diabetes would not have been detected without screening.

The most significant clinical implication of the present study is that patients with the same duration of diabetes may face varying risks of diabetic retinopathy depending on how their diabetes was diagnosed. History taking about how diabetes was diagnosed may be important to manage the risk stratification for diabetic retinopathy appropriately. Physicians should consider a higher risk of diabetic retinopathy and carefully screen for complications when patients are initially diagnosed with diabetes without recent health checkup results.

It is important to acknowledge that the difference in risks according to how diabetes was diagnosed may partly be explained by the lead time bias^11^. For example, if a patient diagnosed with diabetes due to higher HbA1c levels detected at a recent health checkup had not participated in the health checkup, the patient would not have sought medical attention, and their diagnosis of diabetes would have been delayed until the next opportunity. This hypothetical delay can be seen as a lead time, which may occur even if the course of the disease, from the actual onset of diabetes to retinopathy, remains unchanged as a result of the health checkup. Therefore, we cannot definitively conclude that health checkups will lower the risk of diabetic retinopathy. Nevertheless, proper glycemic control and management of hypertension and dyslipidemia are important to reduce the risk of diabetic retinopathy. Early detection of diabetes enables appropriate care, and health checkups may reduce the risk of diabetic retinopathy. A previous nonrandomized controlled trial showed that diabetes screening and cardiovascular risk assessment were associated with lower mortality and cardiovascular events among patients diagnosed with diabetes through screening^12^.

Harris et al. analyzed the S-curve representing the incidence of retinopathy. They calculated the cumulative incidence of retinopathy for each time course from the diagnosis of type 2 diabetes using the USA and Australia cohorts. They determined the intersection of the approximate straight line with the *x*-axis. They concluded that the onset of retinopathy begins occurring 4–7 years before the diagnosis of diabetes (onset–diagnosis gap)^13^. Similarly, Porta et al.^14^ performed a similar analysis using a model that imposed quadratic and S-curve conditions on the model, which estimated the interval between the onset and diagnosis of diabetes to be approximately 6 years. These findings prompted us to investigate whether differences in the cumulative incidence of retinopathy after diabetes diagnosis could indicate variations in the onset–diagnosis gap. Further studies are warranted to explore differences in the onset–diagnosis gap among the groups described in our study. However, it is important to note that the glycemic control during the onset–diagnosis gap is heterogeneous, while the glycemic level soon after the onset is, by definition, just above the threshold of diabetes diagnosis (HbA1c ≥ 6.5%). In contrast, diabetes may worsen during more prolonged undiagnosed periods. The impact of the duration of undiagnosed diabetes on future complications may also differ from that of the diagnosed diabetes duration, depending on the level of care, including self-care.

As demonstrated in Supplementary Fig. S3, the relationship between sex or employment status (employee/dependent) and the outcome events appeared to be modified by how diabetes was diagnosed. In Japan, the participation rate of Specific Health Checkups among dependents has been consistently lower than that among employees. The higher incidence rate among dependents in Group 2 may be attributed to particularly prolonged undiagnosed periods of diabetes among this population. Because Group 5 was limited to the participants of recent health checkups, the duration between the actual incidence of diabetes and the clinical diagnosis of diabetes may not have differed between employees and dependents.

The present study has several limitations. First, we used the intervention for diabetic retinopathy as a proxy for the incidence of diabetic retinopathy requiring intervention. A gap exists between the incidence of diabetic retinopathy and the treatment received, as well as an onset–diagnosis gap of diabetes examined in the present study. To decrease the detection bias, we performed a sensitivity analysis of patients who underwent an eye exam between 6 months before and 1 year after the diabetes diagnosis. Second, we categorized patients with diabetes based on how their diabetes was diagnosed using claims and health checkup data. We excluded those whose timing of diagnosis could not be distinguished. For example, we excluded those whose first HbA1c measurement was observed > 30 days from diagnosis (disease name of first diabetes or use of antidiabetic medication). In contrast, many patients were diagnosed with diabetes following repeated measurements of HbA1c. More detailed information is required to investigate the risks for these patients.

In conclusion, our study revealed heterogeneous future risks of diabetic retinopathy requiring treatment based on how diabetes was diagnosed. Particularly, patients who started their antidiabetic medication promptly after diagnosis without a recent health checkup faced a higher risk. History taking about how diabetes was diagnosed, in addition to diabetes duration, may be important to manage the risk stratification for diabetic retinopathy appropriately.

## Supplementary Information

Supplementary Information.

## Data Availability

The data that support the findings of this study are available from JMDC Inc., but restrictions apply to the availability of these data, which were used under license for the current study, and so are not publicly available. Data are, however, available from the corresponding author upon even reasonable request and with permission of JMDC Inc.
