# Supplementary material for: Different incidences of diabetic retinopathy requiring treatment since diagnosis according to the course of diabetes diagnosis: a retrospective cohort study
Source: Sci Rep. 2023 Jun 29;13:10527. doi: 10.1038/s41598-023-37551-w (PMC10310829; doi:10.1038/s41598-023-37551-w)
Supplement: Supplementary file 1 — Supplementary Information. [file 41598_2023_37551_MOESM1_ESM.docx]

**Supplementary information**

**Supplementary Table S1. Definition of each variable.**

**Supplementary Table S2. Number of study participants and those excluded due to experiencing outcome event >30 days before the date of diabetes diagnosis for each group.**

**Supplementary Table S3. Incidence rates of diabetic retinopathy requiring treatment.**

**Supplementary Table S4. The RECORD guideline checklist.**

**Supplementary Figure S1. Outline of patient selection and data collection.**

**Supplementary Figure S2. Cumulative incidence of treatment-requiring diabetic retinopathy since diabetes diagnosis by characteristics of patients.**

**Supplementary Figure S3. Cumulative incidence of treatment-requiring diabetic retinopathy since diabetes diagnosis by characteristics of patients among Groups 2, 5, and 6.**

**Supplementary References. References cited in the Supplementary Online Content.**

**Supplementary Table S1. Definition of each variable.**

| **Clinical diagnosis of diabetes** | |
| --- | --- |
| Data source | - The medical claims data |
| Definition | - Either the disease name of diabetes or antidiabetic medication. - The disease name of diabetes was defined as E10-14 of ICD-10. - Disease name has each “start date of treatment” information determined by a physician on medical claims; we defined the date of the first diagnosis of diabetes based on the information. - Antidiabetic medication was defined with the A10 code of the ATC classification system, excluding A10X (aldose reductase inhibitor) and Voglibose 0.2mg tablet (possibly used for impaired glucose tolerance). |
| **Treatment-requiring diabetic retinopathy** | |
| Data source | - The medical claims data |
| Definition | - Procedure code of K276 (laser photocoagulation), G016 (intraocular injection), K280, K280-2, or K281 (vitrectomy). - K280, K280-2, or K281 (vitrectomy) was used as an alternate outcome variable in a sensitivity analysis. |
| **Recent health checkup** | |
| Data source | - The health checkup data |
| Definition | - The health checkup within 6 months before the time zero was used. Especially, the health checkup should have HbA1c measurement. - The value of HbA1c was also used for categorization: <6.5% or >= 6.5%. |
| **Age, gender, employees/dependent variable** | |
| Data source | - The ledger of beneficiary |
| Definition | - Age was calculated from the month of birth and the date of diabetes diagnosis; accurately, the age was on the last day in the previous month of the diabetes diagnosis. - Gender, employees/dependent variables were also available in the ledger of the beneficiary. |
| **Type of diabetes** | |
| Data source | - The medical claims data |
| Definition | - Type 1 diabetes was defined as having E10 of ICD-10 within one month from the clinical diagnosis of diabetes. - Type 2 and others were defined as having E11-E14 of ICD-10 and not having E10 within one month from the clinical diagnosis of diabetes. |
| **Hypertension drugs** | |
| Data source | - The medical claims data |
| Definition | - Having both a drug used for hypertension and a hypertension disease name in the same month. - Drugs used for hypertension: C02, C03, C04, C07, C08, C09, and C10BX in the ATC Classification System by WHO collaborating center for Drug Statistics Methodology ATC/DDD Index 2019 (https://www.whocc.no/atc_ddd_index/) - Hypertension disease name: I10-I15 of ICD-10. - We included information observed from 6 months before and 1 months after the time zero. |
| **Dyslipidemia drugs** | |
| Data source | - The medical claims data |
| Definition | - Drugs used for hypertension: C10 in the ATC Classification System by WHO collaborating center for Drug Statistics Methodology ATC/DDD Index 2019 (https://www.whocc.no/atc_ddd_index/) - We included information observed from 6 months before and 1 months after the time zero. |
| **Charlson comorbidity index** | |
| Data source | - The medical claims data |
| Definition | - Charlson comorbidity index updated by Quan et al.^1^ - We included congestive heart failure, dementia, chronic pulmonary disease, rheumatologic disease, mild liver disease, hemiplegia or paraplegia, renal disease, any malignancy including leukemia and lymphoma, moderate or severe liver disease, and metastatic solid tumor. The ICD-10 codes for each disease are as shown in Sundararajan et al.^2^ - We did not include diabetes with/without complications because all of them had diabetes and AIDS/HIV because the disease name information for AIDS/HIV was intentionally excluded from the database for a privacy reason. - We included information observed from 6 months before and 1 months after the time zero. |
| **Eye exams** | |
| Data source | - The medical claims data |
| Definition | - Procedure code of D255, D255-2, D256, D256-2, D256-3, and D257. |

**Supplementary Table S2. Number of study participants and those excluded as they experienced outcome event >30 days before the date of diabetes diagnosis for each group.**

|  | Total | Group 1 | Group 2 | Group 3 | Group 4 | Group 5 | Group 6 | P-value |
| --- | --- | --- | --- | --- | --- | --- | --- | --- |
| Study participants | 126,696  (99.2%) | 69,886  (99.2%) | 5,029  (99.6%) | 36,975  (99.1%) | 581  (99.0%) | 9,843  (99.4%) | 4,382  (99.4%) | .002 |
| Those who were excluded | 993  (0.8%) | 552  (0.8%) | 19  (0.4%) | 324  (0.9%) | 6  (1.0%) | 64  (0.6%) | 28  (0.6%) |  |

P-value is calculated using a chi-squared test.

**Supplementary Table S3. Incidence rates of diabetic retinopathy requiring treatment.**

|  | Number of subjects at baseline | Person-years | Number of cases | Incidence/1,000 person-years (95% CI) | P-value |
| --- | --- | --- | --- | --- | --- |
| Total | 126,696 | 294,611 | 1603 | 5.44 (5.18–5.71) |  |
| Groups |  |  |  |  |  |
| Group 1 | 69,886 | 161,308 | 866 | 5.37 (5.02–5.74) | <.001 |
| Group 2 | 5,029 | 11,322 | 195 | 17.22 (14.97–19.82) |  |
| Group 3 | 36,975 | 86,680 | 312 | 3.60 (3.22–4.02) |  |
| Group 4 | 581 | 1,403 | 3 | 2.14 (0.69–6.63) |  |
| Group 5 | 9,843 | 24,003 | 135 | 5.62 (4.75–6.66) |  |
| Group 6 | 4,382 | 9,894 | 92 | 9.30 (7.58–11.41) |  |
| Age category at baseline (years) |  |  |  |  |  |
| < 30 | 7,378 | 14,813 | 22 | 1.49 (0.98–2.26) | <.001 |
| 30-39 | 18,101 | 45,146 | 87 | 1.93 (1.56–2.38) |  |
| 40-49 | 37,115 | 95,323 | 375 | 3.93 (3.55–4.35) |  |
| 50-59 | 42,832 | 100,156 | 724 | 7.23 (6.72–7.77) |  |
| 60-69 | 18,829 | 35,508 | 346 | 9.74 (8.77–10.83) |  |
| >= 70 | 2,441 | 3,645 | 49 | 13.44 (10.16–17.79) |  |
| Gender |  |  |  |  |  |
| Male | 78,775 | 188,216 | 918 | 4.88 (4.57–5.20) | <.001 |
| Female | 47,921 | 106,395 | 685 | 6.44 (5.97–6.94) |  |
| Employee/Dependent |  |  |  |  |  |
| Employee | 93,197 | 218,140 | 1,018 | 4.67 (4.39–4.96) | <.001 |
| Dependent | 33,499 | 76,471 | 585 | 7.65 (7.05–8.30) |  |
| Type of diabetes |  |  |  |  |  |
| Type 1 diabetes | 601 | 1,394 | 17 | 12.19 (7.58–19.61) | <.001 |
| Type 2 and others | 126,095 | 293,217 | 1,586 | 5.41 (5.15–5.68) |  |
| Hypertension drugs |  |  |  |  |  |
| No | 95,068 | 224,027 | 1,020 | 4.55 (4.28–4.84) | <.001 |
| Yes | 31,628 | 70,584 | 583 | 8.26 (7.62–8.96) |  |
| Dyslipidemia drugs |  |  |  |  |  |
| No | 101,468 | 236,122 | 1,252 | 5.30 (5.02–5.60) | .04 |
| Yes | 25,228 | 58,489 | 351 | 6.00 (5.41–6.66) |  |
| CCI of >= 3 |  |  |  |  |  |
| No | 108,297 | 255,534 | 1,362 | 5.33 (5.05–5.62) | .12 |
| Yes | 18,399 | 39,077 | 241 | 6.17 (5.44–7.00) |  |

^a^ Charlson comorbidity index.

P-values are calculated using log-rank tests.

**Supplementary Table S4. The RECORD statement – checklist of items, extended from the STROBE statement that should be reported in observational studies using routinely collected health data.**

|  | **Item No.** | **STROBE items** | **Location in manuscript where items are reported** | **RECORD items** | **Location in manuscript where items are reported** |
| --- | --- | --- | --- | --- | --- |
| **Title and abstract** | | | | | |
|  | 1 | (a) Indicate the study’s design with a commonly used term in the title or the abstract (b) Provide in the abstract an informative and balanced summary of what was done and what was found | (a) Abstract  (b) Abstract | RECORD 1.1: The type of data used should be specified in the title or abstract. When possible, the name of the databases used should be included.  RECORD 1.2: If applicable, the geographic region and timeframe within which the study took place should be reported in the title or abstract.  RECORD 1.3: If linkage between databases was conducted for the study, this should be clearly stated in the title or abstract. | Abstract  Abstract  N/A |
| **Introduction** | | | | | |
| Background rationale | 2 | Explain the scientific background and rationale for the investigation being reported | Introduction |  |  |
| Objectives | 3 | State specific objectives, including any prespecified hypotheses | Introduction |  |  |
| **Methods** | | | | | |
| Study Design | 4 | Present key elements of study design early in the paper | Data sources and study population |  |  |
| Setting | 5 | Describe the setting, locations, and relevant dates, including periods of recruitment, exposure, follow-up, and data collection | Data sources and study population |  |  |
| Participants | 6 | *(a) Cohort study* - Give the eligibility criteria, and the sources and methods of selection of participants. Describe methods of follow-up  *Case-control study* - Give the eligibility criteria, and the sources and methods of case ascertainment and control selection. Give the rationale for the choice of cases and controls  *Cross-sectional study* - Give the eligibility criteria, and the sources and methods of selection of participants  *(b) Cohort study* - For matched studies, give matching criteria and number of exposed and unexposed  *Case-control study* - For matched studies, give matching criteria and the number of controls per case | (a) Data sources and study population  eFigure 1  (b) N/A | RECORD 6.1: The methods of study population selection (such as codes or algorithms used to identify subjects) should be listed in detail. If this is not possible, an explanation should be provided.  RECORD 6.2: Any validation studies of the codes or algorithms used to select the population should be referenced. If validation was conducted for this study and not published elsewhere, detailed methods and results should be provided.  RECORD 6.3: If the study involved linkage of databases, consider use of a flow diagram or other graphical display to demonstrate the data linkage process, including the number of individuals with linked data at each stage. | Data sources and study population  (Supplementary Figure S1)  N/A  N/A |
| Variables | 7 | Clearly define all outcomes, exposures, predictors, potential confounders, and effect modifiers. Give diagnostic criteria, if applicable | Measurements | RECORD 7.1: A complete list of codes and algorithms used to classify exposures, outcomes, confounders, and effect modifiers should be provided. If these cannot be reported, an explanation should be provided. | Supplementary Table S1 |
| Data sources/ measurement | 8 | For each variable of interest, give sources of data and details of methods of assessment (measurement).  Describe comparability of assessment methods if there is more than one group | Measurements  eTable 1 |  |  |
| Bias | 9 | Describe any efforts to address potential sources of bias | Statistical analyses |  |  |
| Study size | 10 | Explain how the study size was arrived at | Results  Figure 1 |  |  |
| Quantitative variables | 11 | Explain how quantitative variables were handled in the analyses. If applicable, describe which groupings were chosen, and why | Statistical analyses |  |  |
| Statistical methods | 12 | (a) Describe all statistical methods, including those used to control for confounding  (b) Describe any methods used to examine subgroups and interactions  (c) Explain how missing data were addressed  (d) *Cohort study* - If applicable, explain how loss to follow-up was addressed  *Case-control study* - If applicable, explain how matching of cases and controls was addressed  *Cross-sectional study* - If applicable, describe analytical methods taking account of sampling strategy  (e) Describe any sensitivity analyses | (a) Statistical analyses  (b) Statistical analyses  (a) Statistical analysis  (d) Statistical analyses  (e) Statistical analyses |  |  |
| Data access and cleaning methods |  | .. |  | RECORD 12.1: Authors should describe the extent to which the investigators had access to the database population used to create the study population.  RECORD 12.2: Authors should provide information on the data cleaning methods used in the study. | Data sources and study population  N/A |
| Linkage |  | .. |  | RECORD 12.3: State whether the study included person-level, institutional-level, or other data linkage across two or more databases. The methods of linkage and methods of linkage quality evaluation should be provided. | N/A |
| **Results** | | | | | |
| Participants | 13 | (a) Report the numbers of individuals at each stage of the study (*e.g.*, numbers potentially eligible, examined for eligibility, confirmed eligible, included in the study, completing follow-up, and analyzed)  (b) Give reasons for non-participation at each stage.  (c) Consider use of a flow diagram | (a) Results  Figure 1  (b) Figure 1  (c) Figure 1 | RECORD 13.1: Describe in detail the selection of the persons included in the study (*i.e.,* study population selection) including filtering based on data quality, data availability and linkage. The selection of included persons can be described in the text and/or by means of the study flow diagram. | Data sources and study population  Figure 1 |
| Descriptive data | 14 | (a) Give characteristics of study participants (*e.g.*, demographic, clinical, social) and information on exposures and potential confounders  (b) Indicate the number of participants with missing data for each variable of interest  (c) *Cohort study* - summarize follow-up time (*e.g.*, average and total amount) | (a) |  |  |
| Outcome data | 15 | *Cohort study* - Report numbers of outcome events or summary measures over time  *Case-control study* - Report numbers in each exposure category, or summary measures of exposure  *Cross-sectional study* - Report numbers of outcome events or summary measures | Results  Figure 1 |  |  |
| Main results | 16 | (a) Give unadjusted estimates and, if applicable, confounder-adjusted estimates and their precision (e.g., 95% confidence interval). Make clear which confounders were adjusted for and why they were included  (b) Report category boundaries when continuous variables were categorized  (c) If relevant, consider translating estimates of relative risk into absolute risk for a meaningful time period | (a) Results  (b) N/A  (c) N/A |  |  |
| Other analyses | 17 | Report other analyses done—e.g., analyses of subgroups and interactions, and sensitivity analyses | Results |  |  |
| **Discussion** | | | | | |
| Key results | 18 | Summarize key results with reference to study objectives | Discussions |  |  |
| Limitations | 19 | Discuss limitations of the study, taking into account sources of potential bias or imprecision. Discuss both direction and magnitude of any potential bias | Discussions | RECORD 19.1: Discuss the implications of using data that were not created or collected to answer the specific research question(s). Include discussion of misclassification bias, unmeasured confounding, missing data, and changing eligibility over time, as they pertain to the study being reported. | Discussion |
| Interpretation | 20 | Give a cautious overall interpretation of results considering objectives, limitations, multiplicity of analyses, results from similar studies, and other relevant evidence | Discussions |  |  |
| Generalizability | 21 | Discuss the generalizability (external validity) of the study results | Discussions |  |  |
| **Other Information** | | | | | |
| Funding | 22 | Give the source of funding and the role of the funders for the present study and, if applicable, for the original study on which the present article is based | Acknowledgments |  |  |
| Accessibility of protocol, raw data, and programming code |  | .. |  | RECORD 22.1: Authors should provide information on how to access any supplemental information such as the study protocol, raw data, or programming code. | N/A |

*Reference: Benchimol EI, Smeeth L, Guttmann A, et al. The Reporting of studies Conducted using Observational Routinely-collected health Data (RECORD) Statement. *PLoS Medicine* 2015; in press.

**Supplementary Figure S1. Outline of patient selection and data collection.**

**
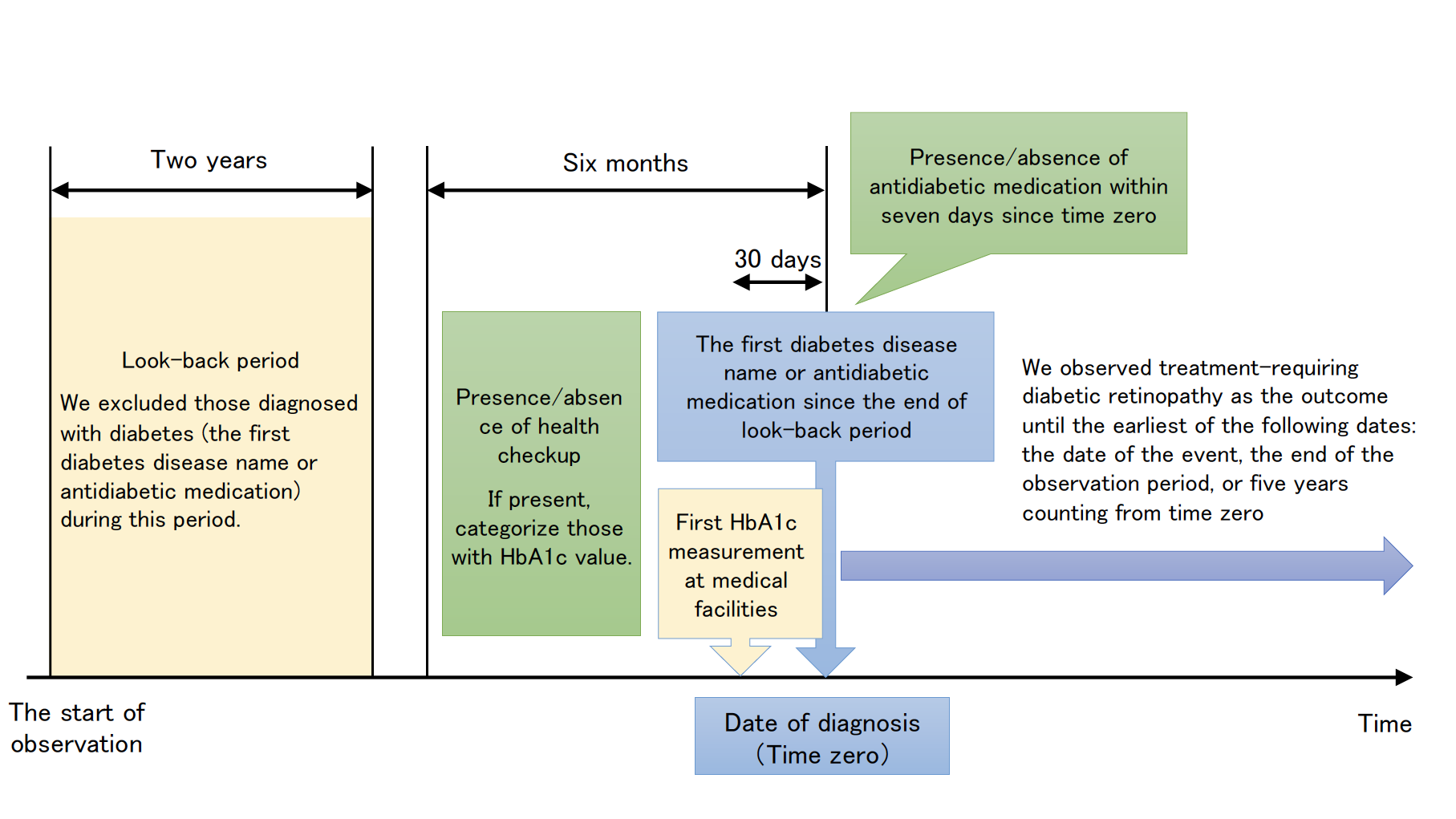
**

First, among those who had diabetes disease name or antidiabetic medication, we excluded those diagnosed with diabetes at medical facilities (excluding health checkups) during the two-year look-back period since the start of the observation for each patient. We identified the first diabetes disease name or antidiabetic medication since the end of the look-back period as the date of diagnosis (time zero). We excluded those whose first HbA1c measurement at medical facilities was not within 30 days from time zero. The existence or absence of a health examination 6 months prior to time zero, the HbA1c value if available, and the presence or absence of antidiabetic medication within seven days since time zero were used to group the participants. A health checkup may or may not be in the lookback period. We followed up with the grouped patients and observed treatment-requiring diabetic retinopathy as the outcome until the earliest of the following date: the date of the event, the end of the observation period, or 5 years from time zero.

**Supplementary Figure S2. Cumulative incidence of treatment-requiring diabetic retinopathy since diabetes diagnosis by characteristics of patients.**

**
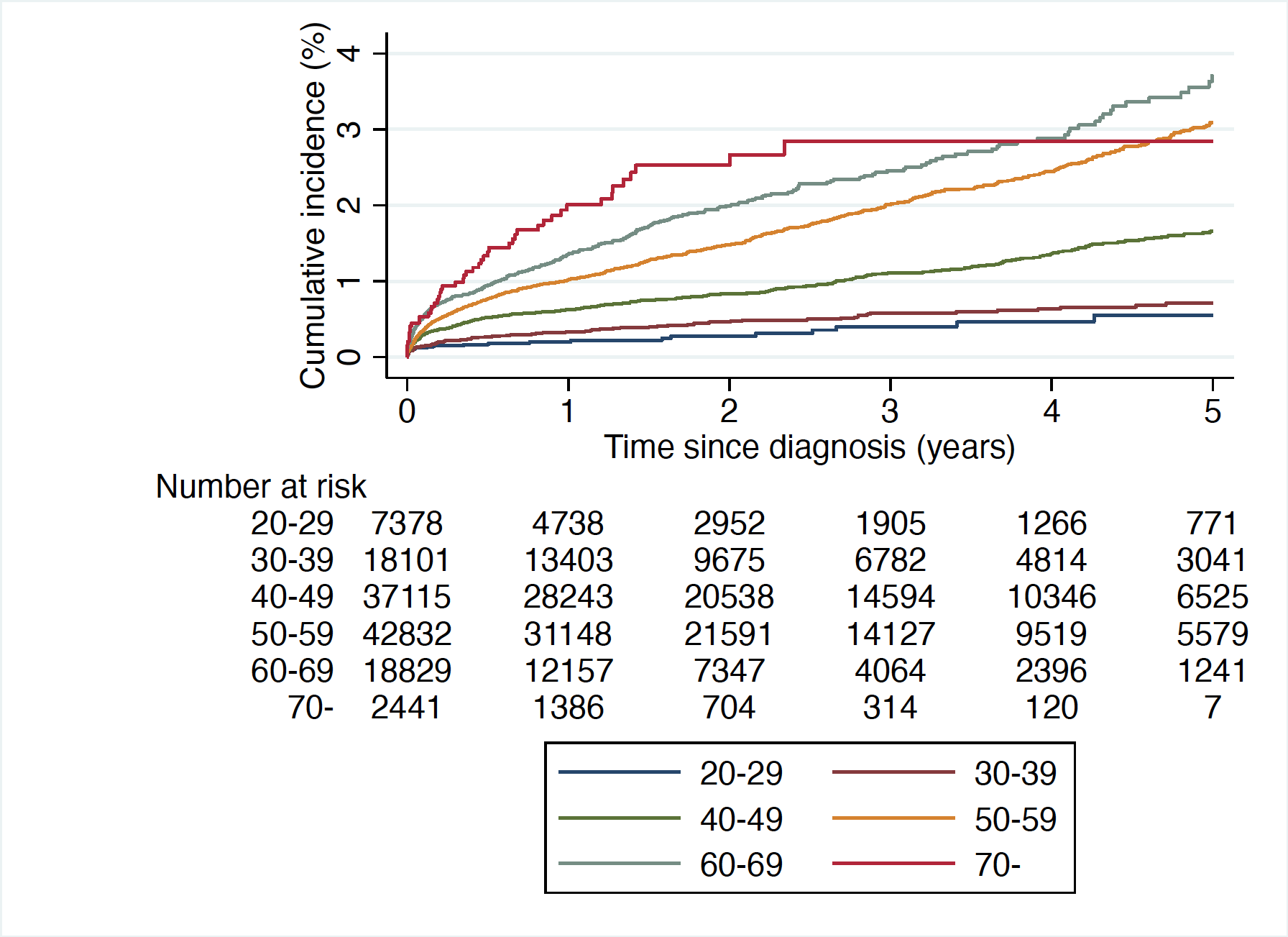
**

1. **By age category at baseline. P < 0.001 by a log-rank test.**

**
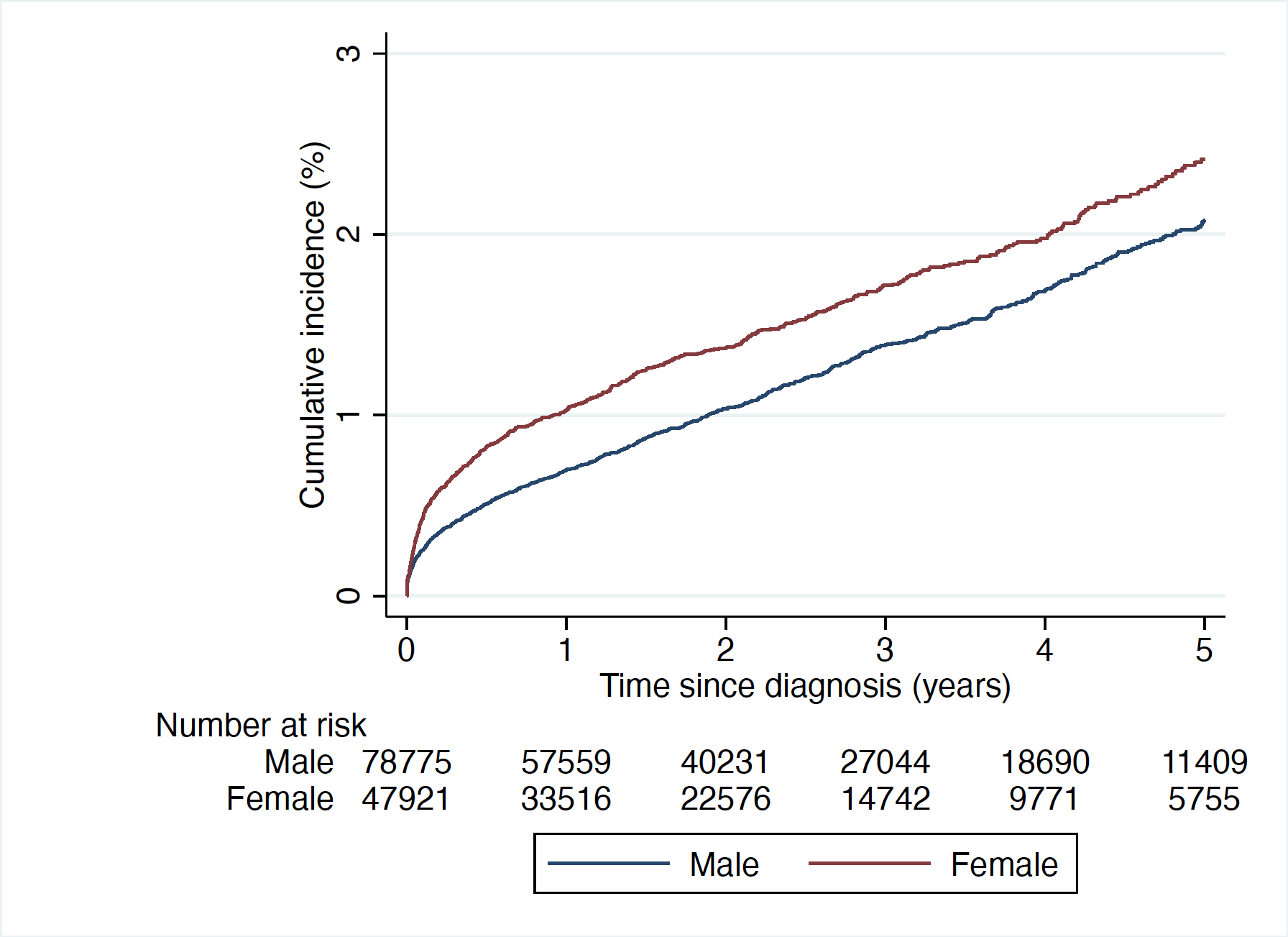
**

1. **By sex. P < 0.001 by a log-rank test.**

**
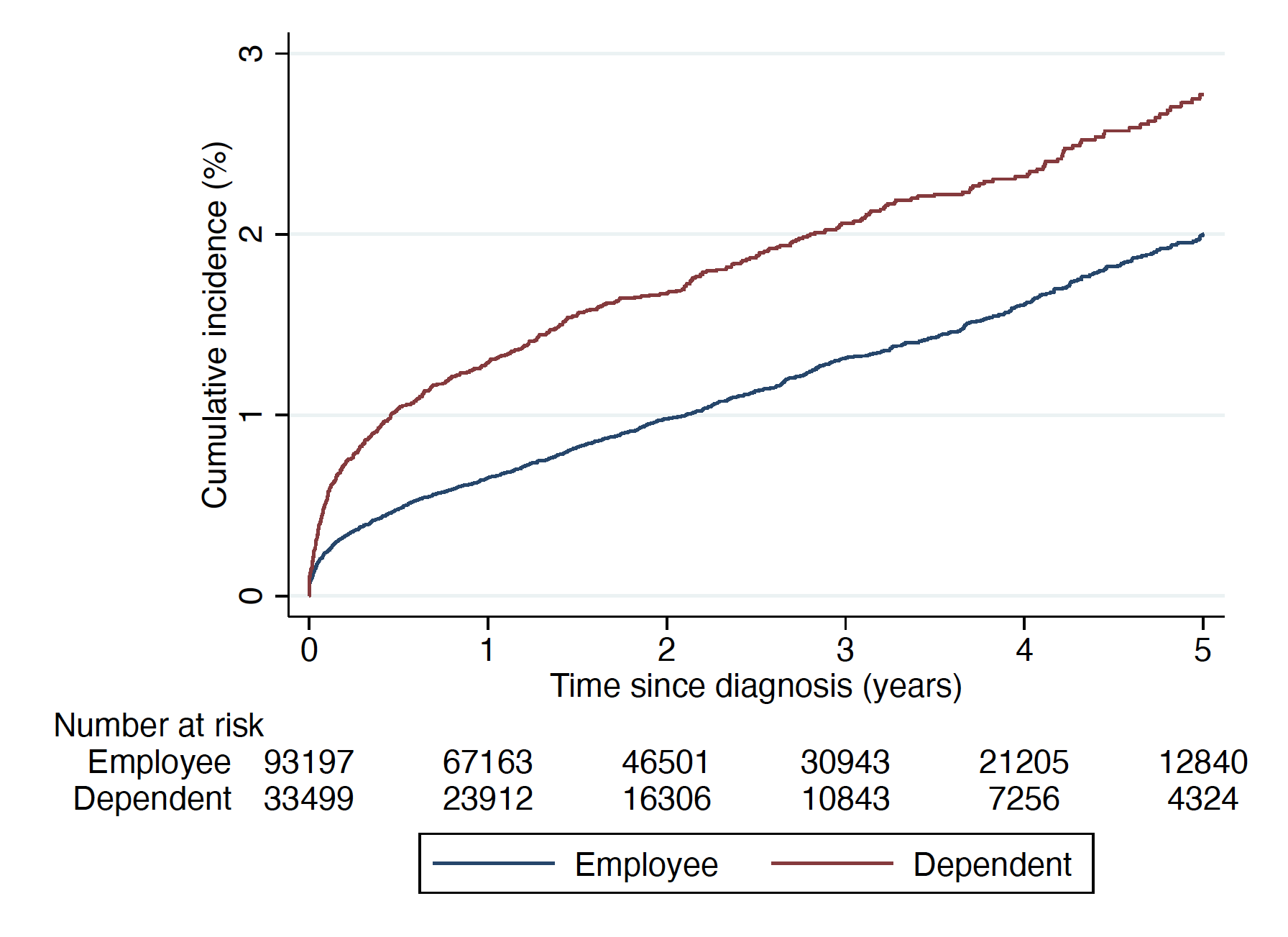
**

1. **By employee/dependent. P < 0.001 by a log-rank test.**

**
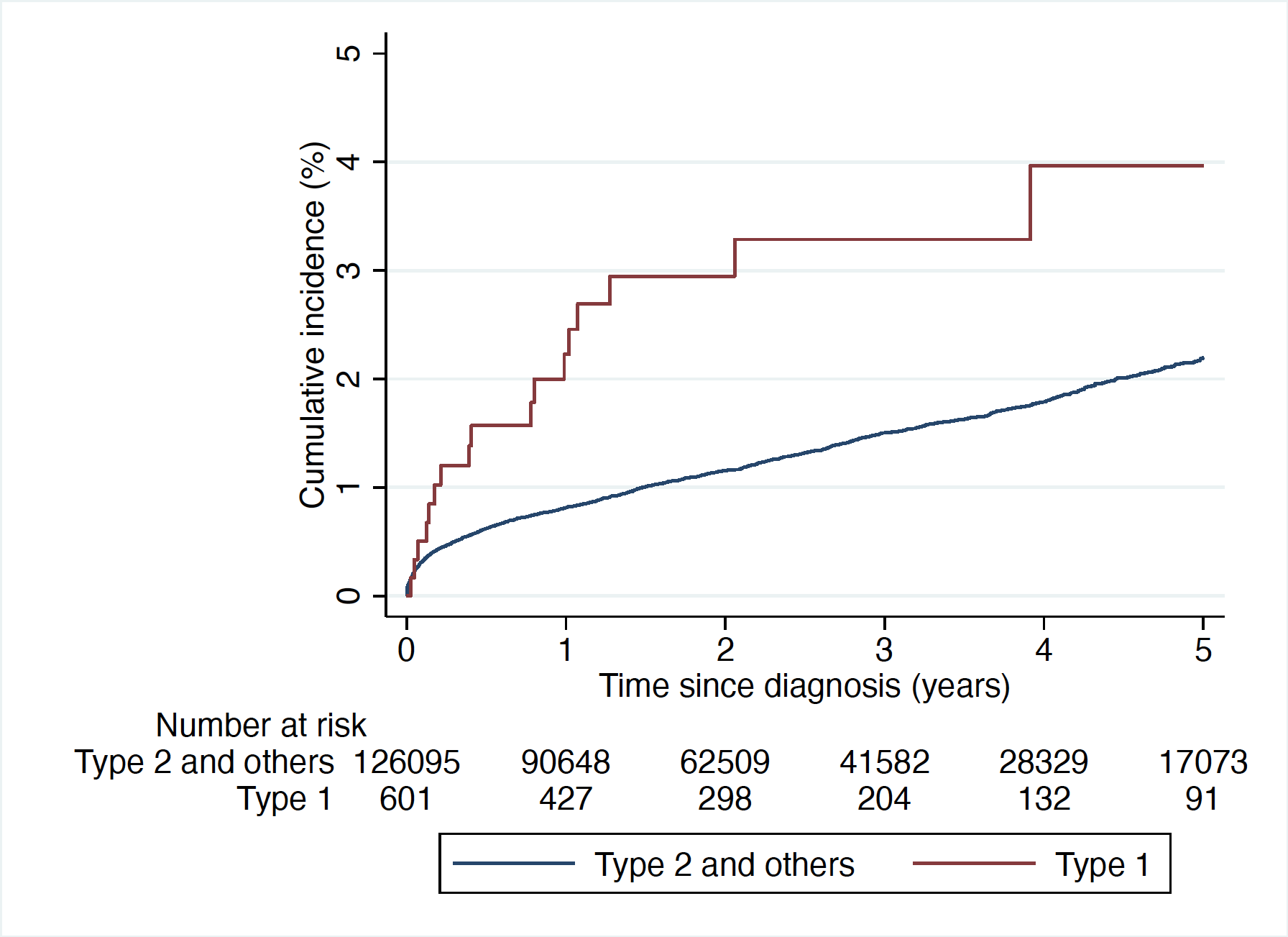
**

1. **By type of diabetes. P < 0.001 by a log-rank test.**

**
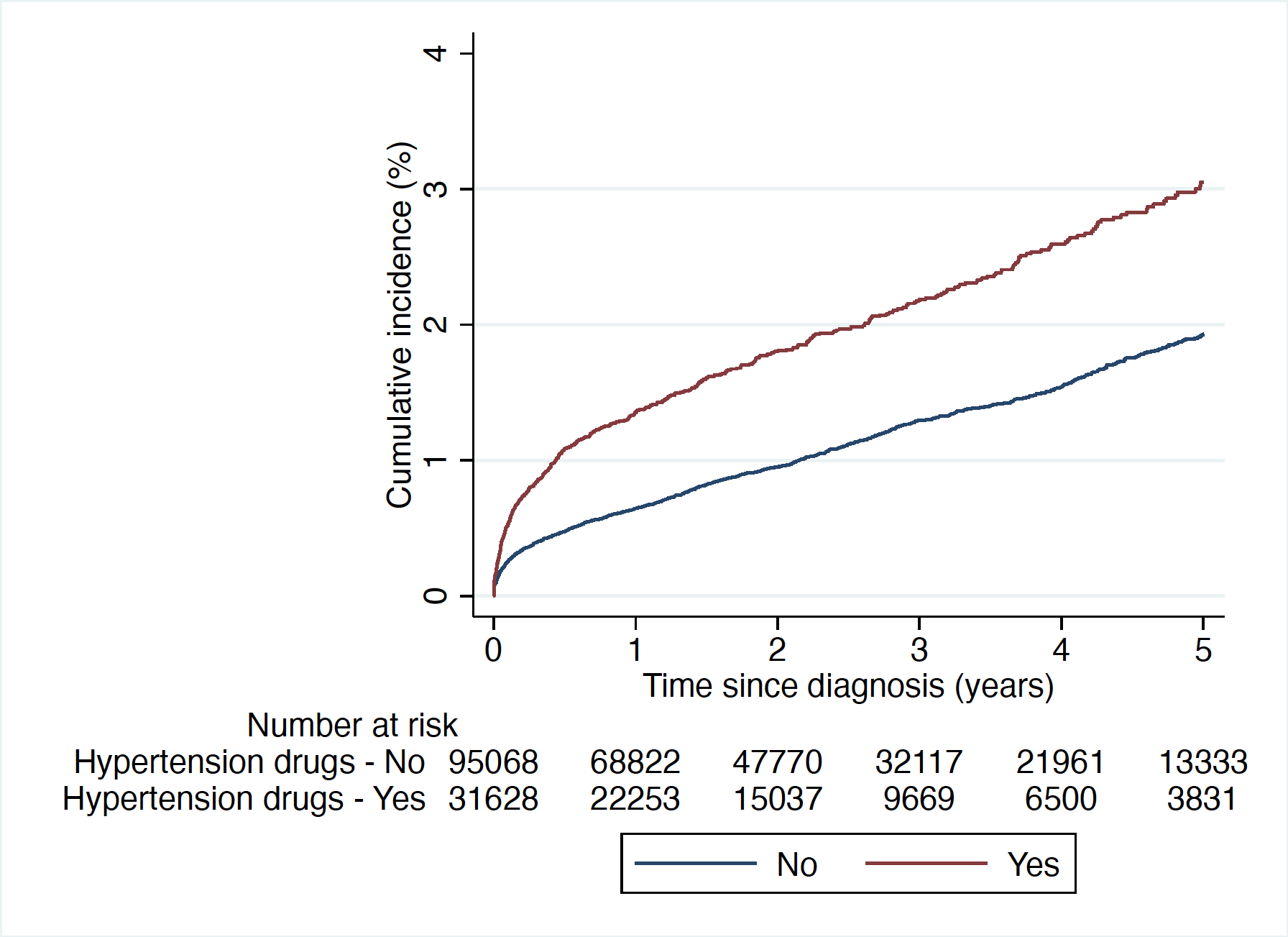
**

1. **By the presence/absence of hypertension drugs. P < 0.001 by a log-rank test.**

**
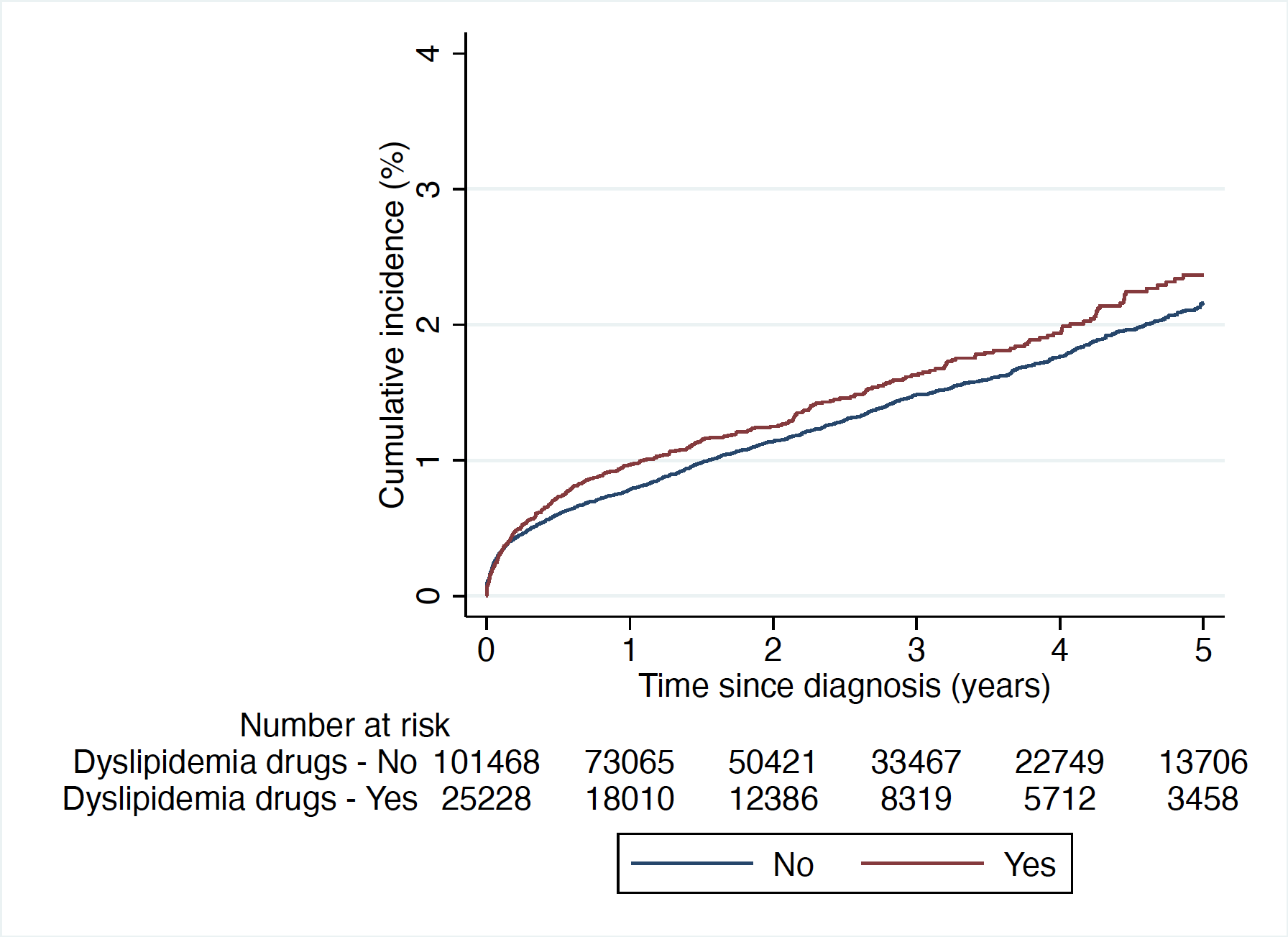
**

1. **By the presence/absence of dyslipidemia drugs. P = 0.04 by a log-rank test.**

**
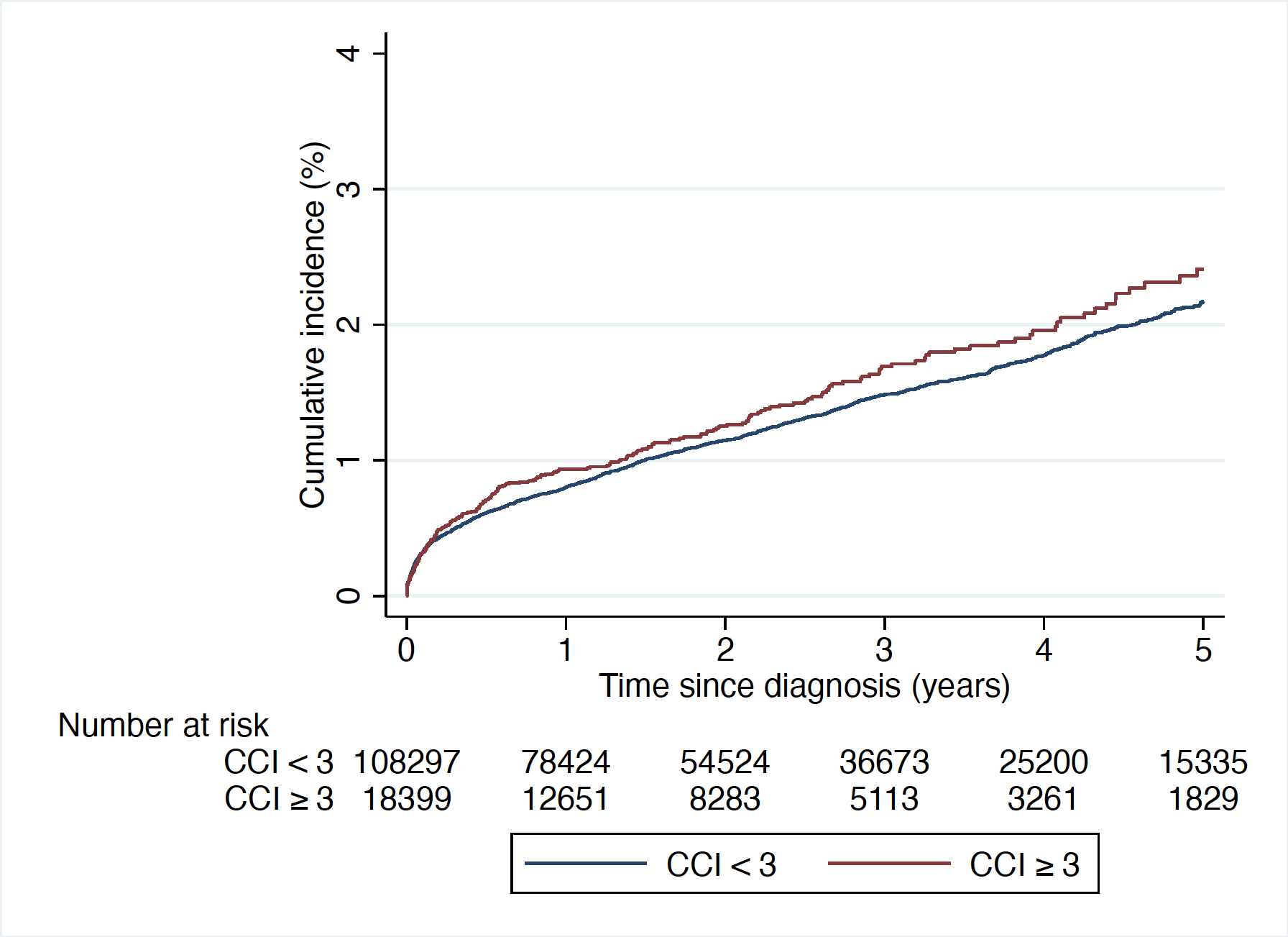
**

1. **By Charlson comorbidity index (CCI) category. P = 0.12 by a log-rank test.**

**.**

**Supplementary Figure S3. Cumulative incidence of treatment-requiring diabetic retinopathy since diabetes diagnosis by characteristics of patients among Groups 2, 5, and 6.**

**
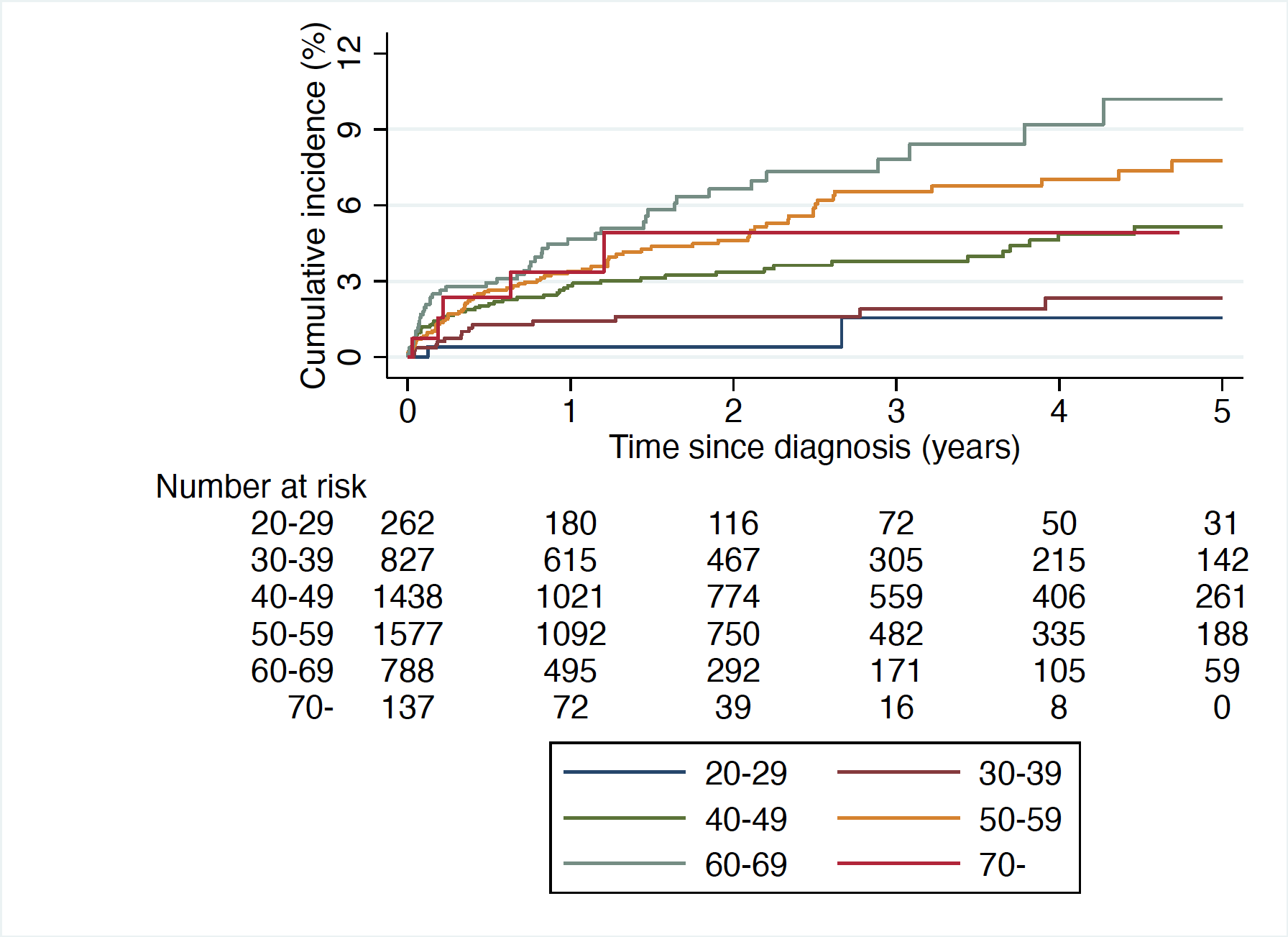
**

1. **By age category at baseline among Group 2. P < 0.001 by a log-rank test.**

**
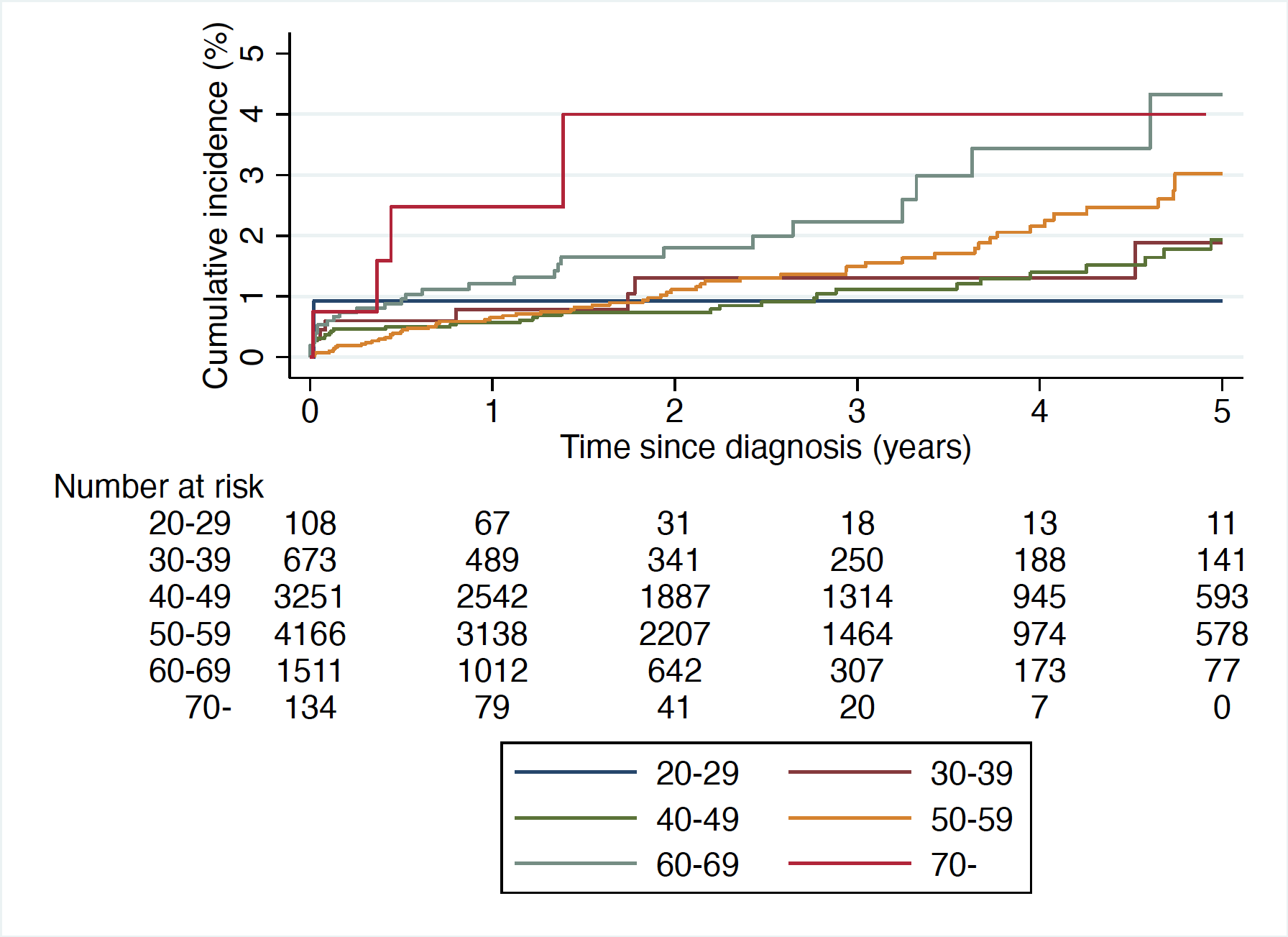
**

1. **By age category at baseline among Group 5. P = 0.005 by a log-rank test.**

**
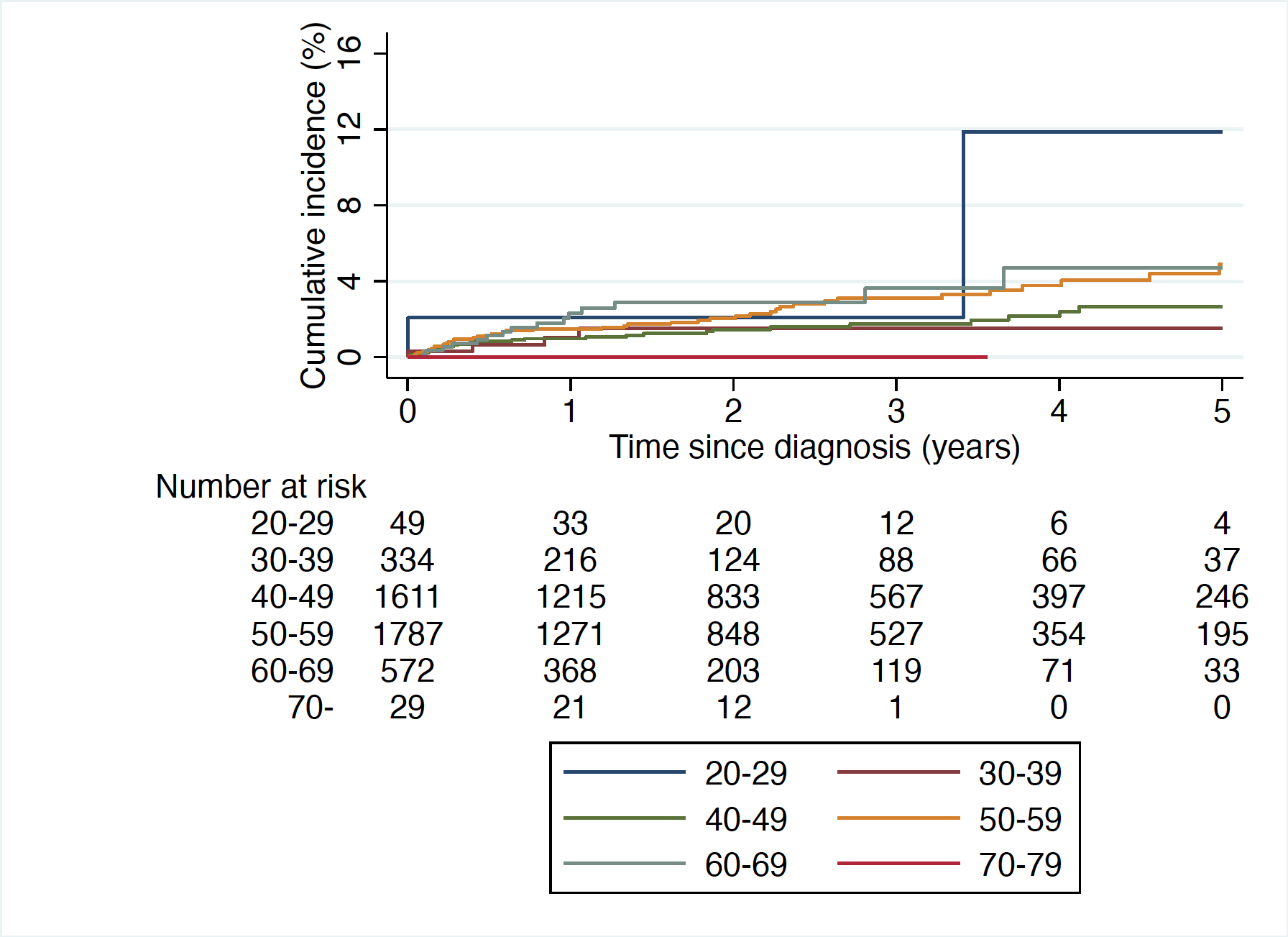
**

1. **By age category at baseline among Group 6. P = 0.12 by a log-rank test.**

**
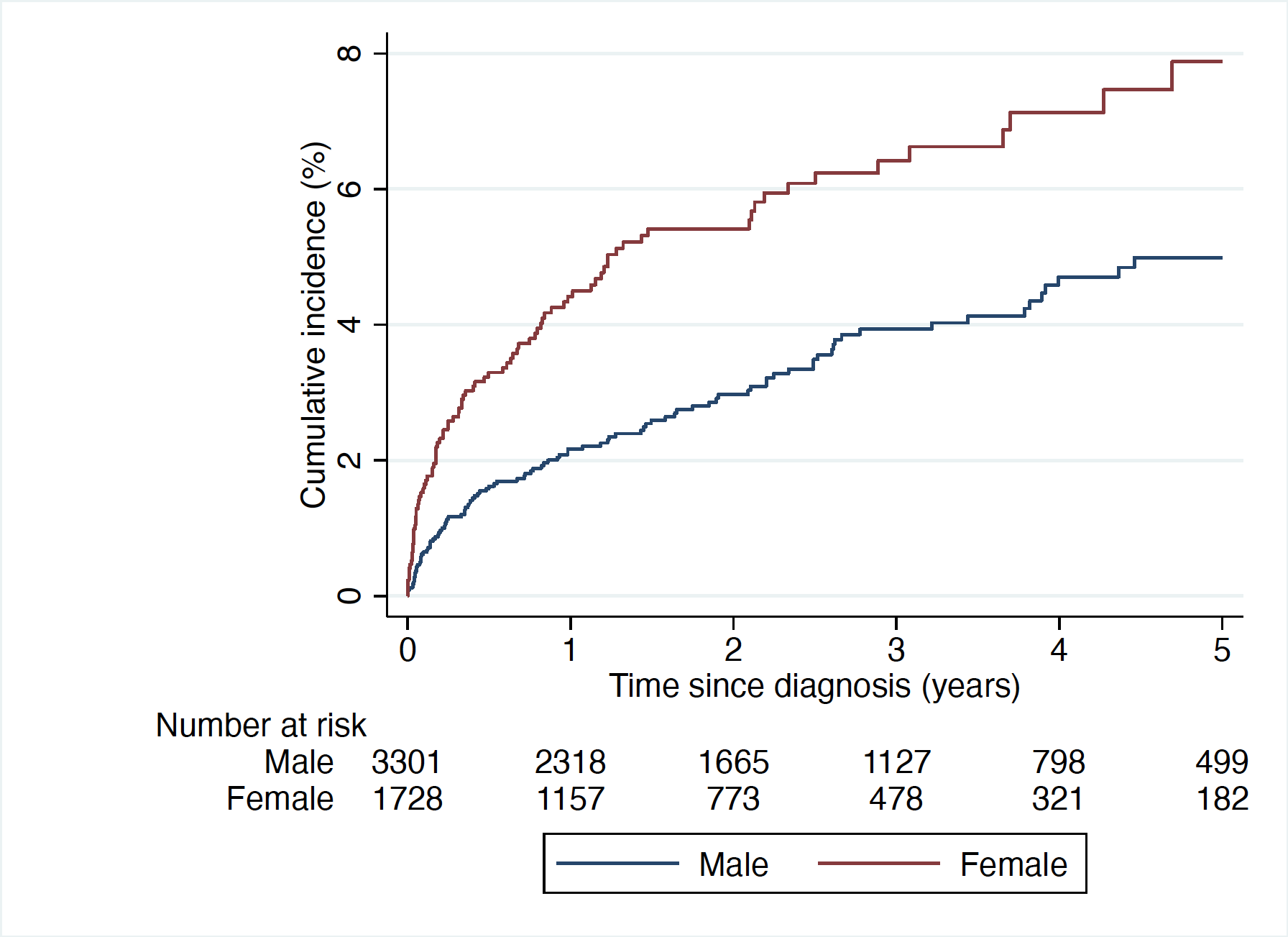
**

1. **By sex among Group 2. P < 0.001 by a log-rank test.**

**
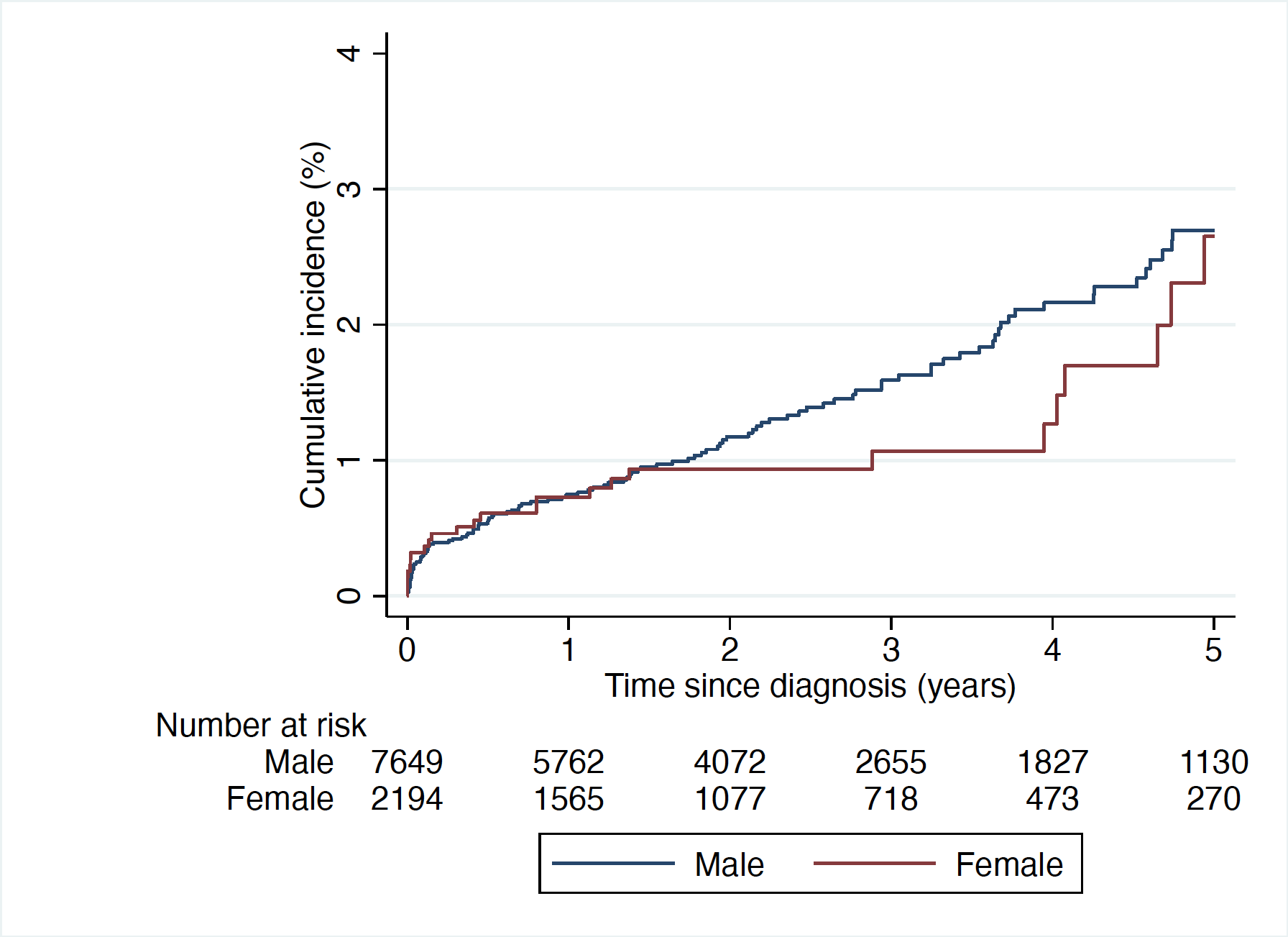
**

1. **By sex among Group 5. P = 0.44 by a log-rank test.**

**
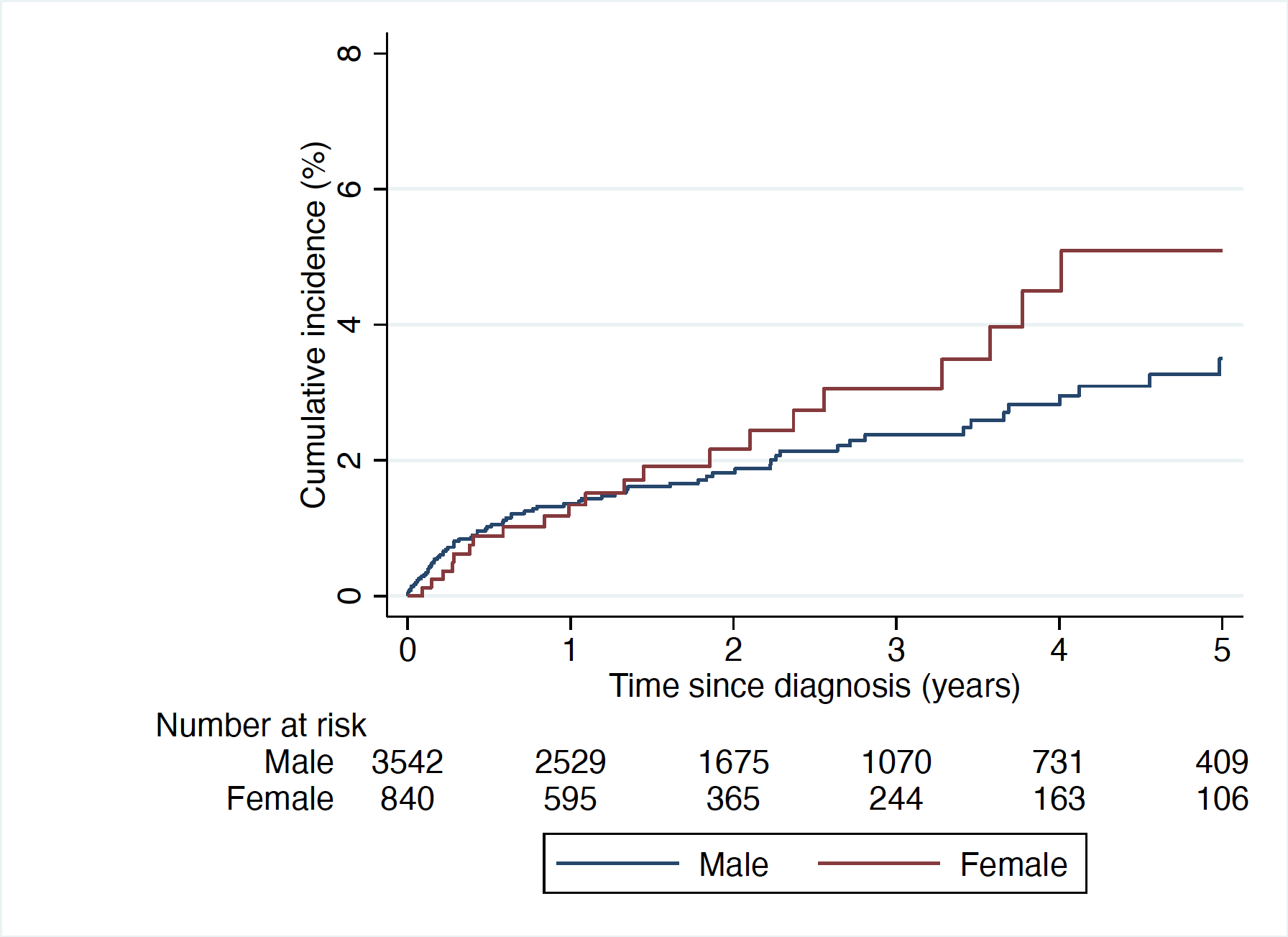
**

1. **By sex among Group 6. P = 0.32 by a log-rank test.**

**
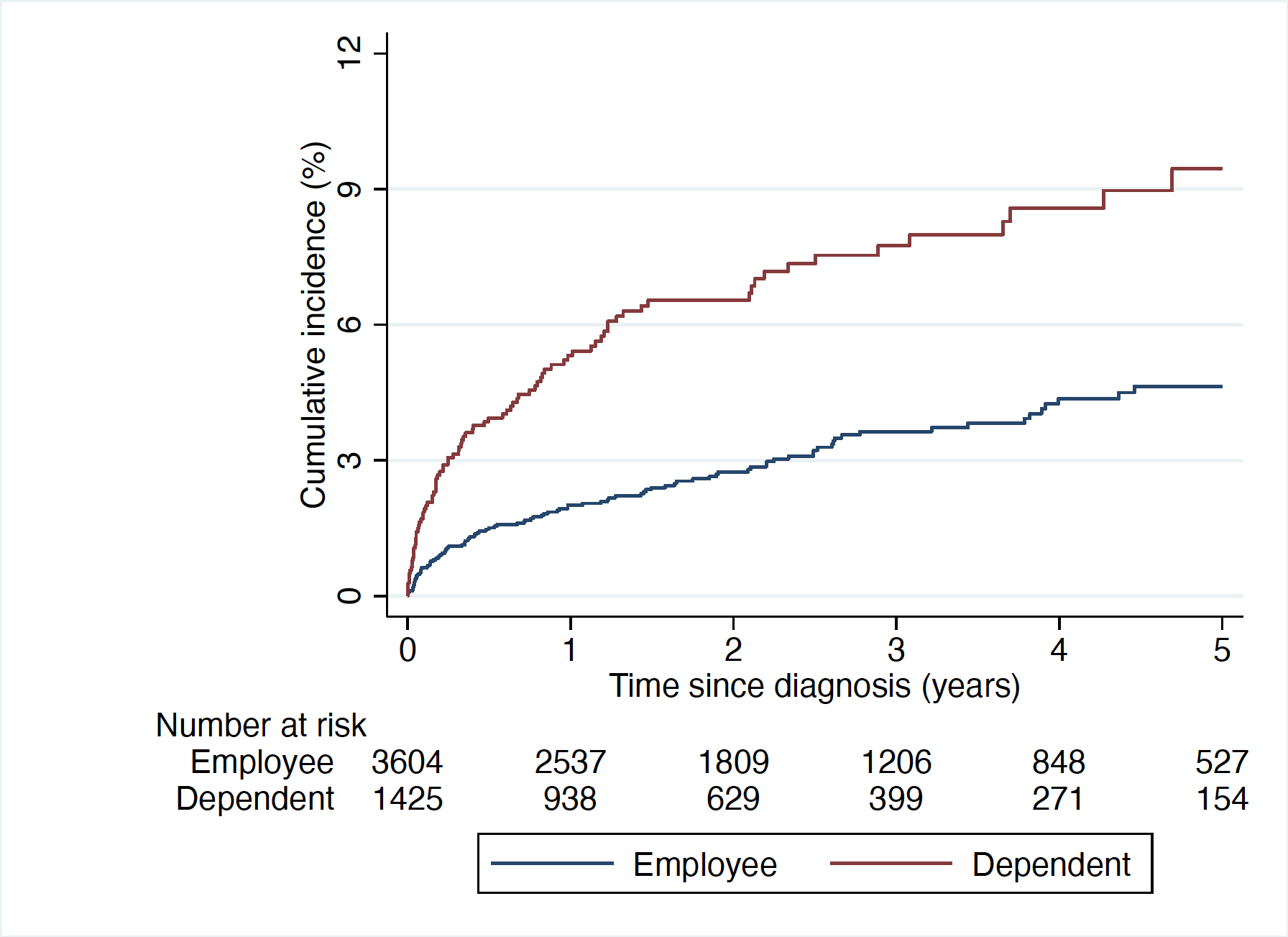
**

1. **By employee/dependent among Group 2. P < 0.001 by a log-rank test.**

**
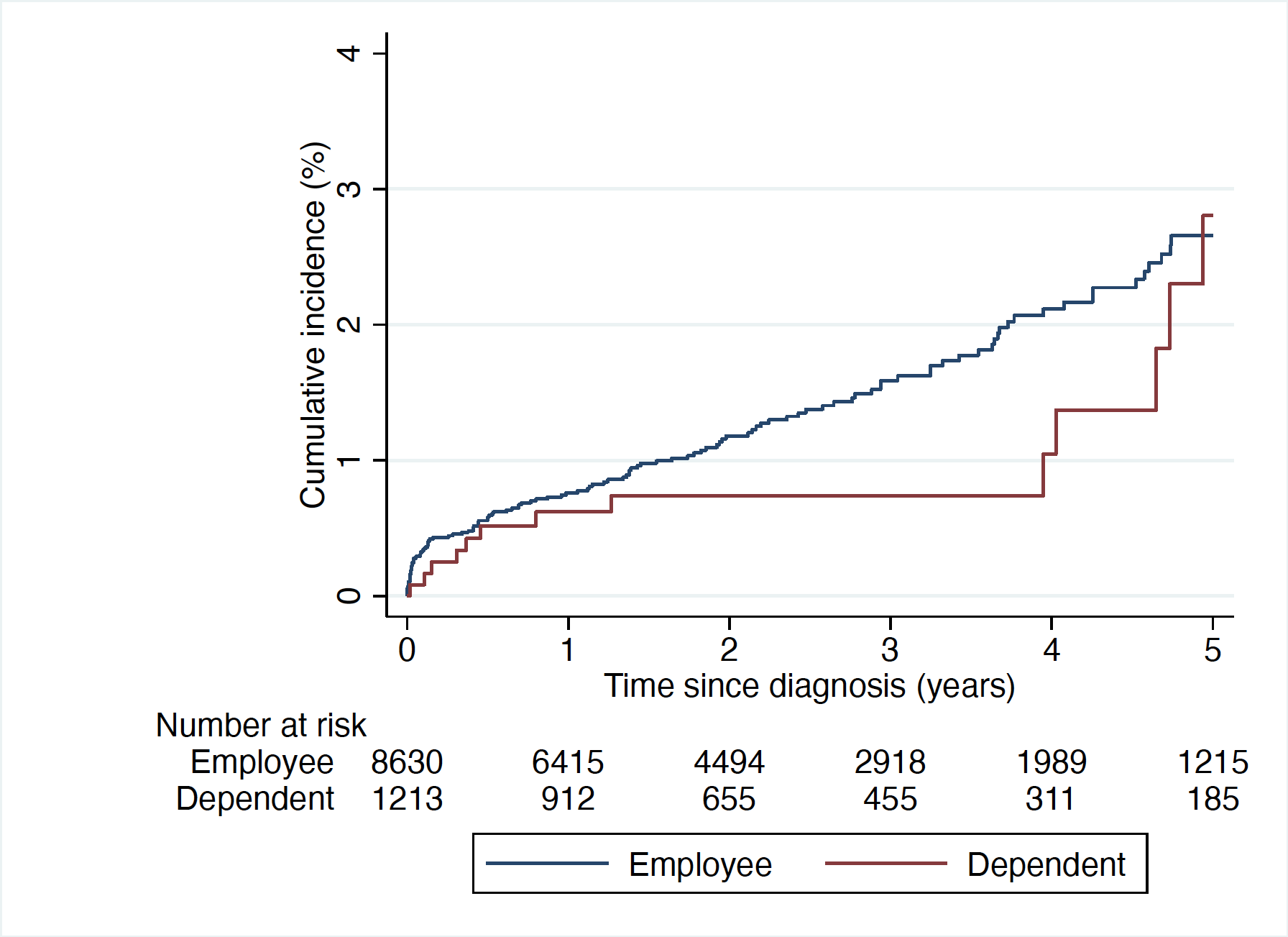
**

1. **By employee/dependent among Group 5. P =0.31 by a log-rank test.**

**
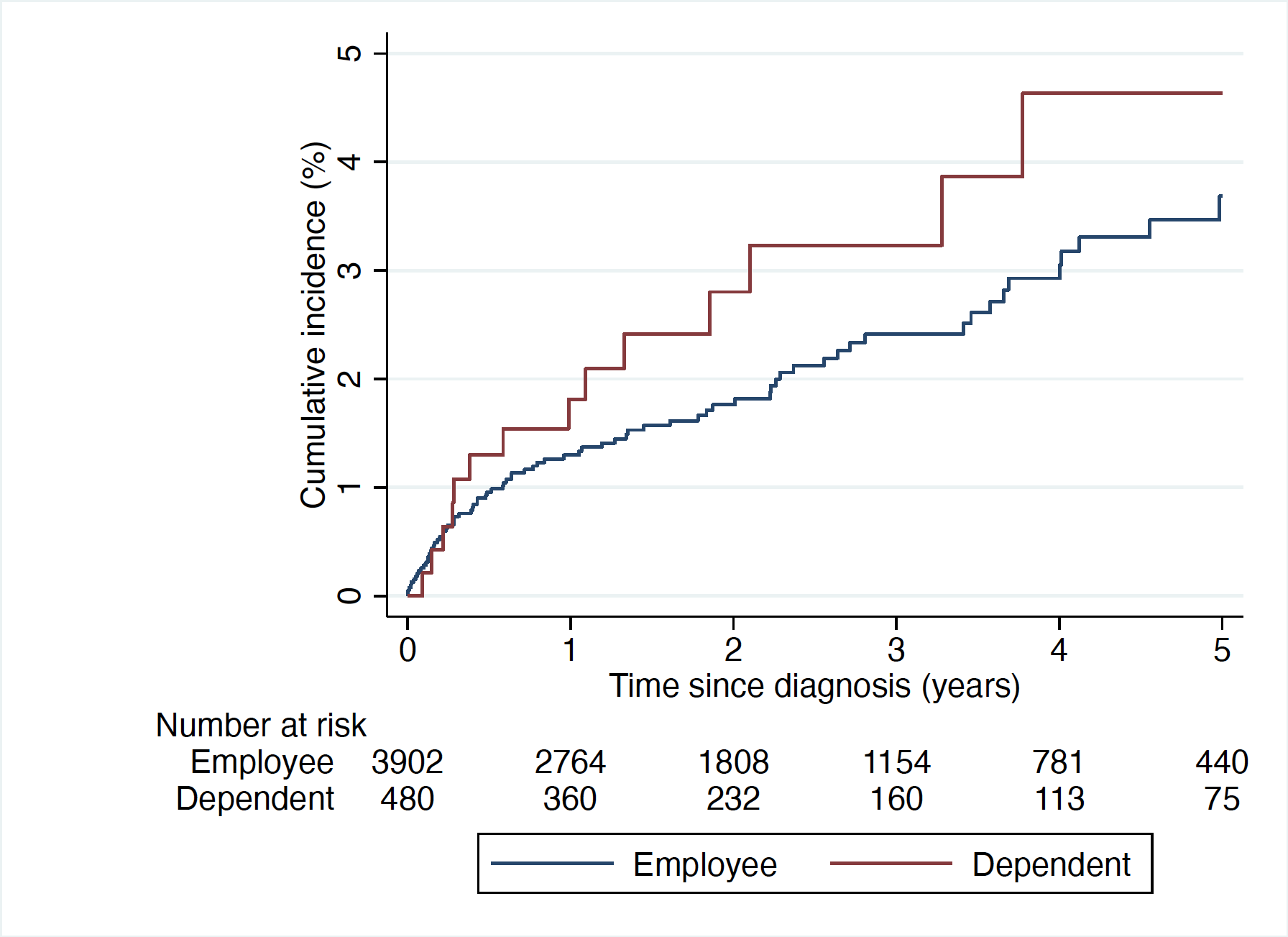
**

1. **By employee/dependent among Group 6. P =0.24 by a log-rank test.**

**Supplementary References. References cited in the Supplementary information.**

1. Quan H, Li B, Couris CM, et al. Updating and validating the Charlson comorbidity index and score for risk adjustment in hospital discharge abstracts using data from 6 countries. *American journal of epidemiology*. Mar 15 2011;173(6):676-82. doi:10.1093/aje/kwq433

2. Sundararajan V, Henderson T Fau - Perry C, Perry C Fau - Muggivan A, Muggivan A Fau - Quan H, Quan H Fau - Ghali WA, Ghali WA. New ICD-10 version of the Charlson comorbidity index predicted in-hospital mortality. (0895-4356 (Print))
